# Gastrodin attenuates renal injury and collagen deposition *via* suppression of the TGF-β1/Smad2/3 signaling pathway based on network pharmacology analysis

**DOI:** 10.3389/fphar.2023.1082281

**Published:** 2023-01-17

**Authors:** Ying Wen, Xiuli Zhang, Lihui Wei, Meizhu Wu, Ying Cheng, Huifang Zheng, Aling Shen, Changgeng Fu, Farman Ali, Linzi Long, Yao Lu, Jiapeng Li, Jun Peng

**Affiliations:** ^1^ Academy of Integrative Medicine, Fujian University of Traditional Chinese Medicine, Fuzhou, China; ^2^ Fujian Key Laboratory of Integrative Medicine in Geriatrics, Fujian University of Traditional Chinese Medicine, Fuzhou, China; ^3^ Fujian Collaborative Innovation Center for Integrative Medicine in Prevention and Treatment of Major Chronic Cardiovascular Diseases, Fuzhou, China; ^4^ Innovation and Transformation Center, Fujian University of Traditional Chinese Medicine, Fuzhou, China; ^5^ National Clinical Research Center for Chinese Medicine Cardiology, Xiyuan Hospital, China Academy of Chinese Medical Sciences, Beijing, China; ^6^ Department of Geriatrics, Xiyuan Hospital, China Academy of Chinese Medical Sciences, Beijing, China; ^7^ Department of Physical Education, Fujian University of Traditional Chinese Medicine, Fuzhou, China

**Keywords:** gastrodin, hypertension, renal fibrosis, TGF-β1/Smad2/3, network pharmacology

## Abstract

**Background:** Gastrodin has been widely used clinically in China as an antihypertensive drug. However, its effect on hypertensive renal injury is yet to be elucidated. The current study aimed to investigate the effects of gastrodin on hypertensive renal injury and its underlying mechanisms by network pharmacology analysis and validation *in vivo* and *in vitro*.

**Methods:** A total of 10 spontaneously hypertensive rats (SHRs) were randomly categorized into the following two groups: SHR and SHR + Gastrodin groups. Wistar Kyoto (WKY) rats were used as the control group (*n* = 5). The SHR + Gastrodin group was intragastrically administered gastrodin (3.5 mg/kg/day), and the rats in both WKY and SHR groups were intragastrically administered an equal amount of double-distilled water for 10 weeks. Hematoxylin-eosin, Masson’s trichrome, and Sirius red staining were used to detect the pathological changes and collagen content in the renal tissues. Network pharmacology analysis was performed to explore its potential targets and related pathways. *In vitro*, the CCK-8 assay was used to determine the cell viability. Immunohistochemistry and western-blotting analyses were employed to assess the protein expression associated with renal fibrosis and transforming growth factor-β1 (TGF-β1) pathway-related proteins in the renal tissues or in TGF-β1-stimulated rat kidney fibroblast cell lines (NRK-49F).

**Results:** Gastrodin treatment attenuates renal injury and pathological alterations in SHRs, including glomerular sclerosis and atrophy, epithelial cell atrophy, and tubular dilation. Gastrodin also reduced the accumulation of collagen in the renal tissues of SHRs, which were confirmed by downregulation of α-SMA, collagen I, collagen III protein expression. Network pharmacology analysis identified TGFB1 and SMAD2 as two of lead candidate targets of gastrodin on against hypertensive renal injury. Consistently, gastrodin treatment downregulated the increase of the protein expression of TGF-β1, and ratios of both p-Smad2/Smad2 and p-Samd3/Smad3 in renal tissues of SHRs. *In vitro*, gastrodin (25–100 μM) treatment significantly reversed the upregulation of α-SMA, fibronectin, collagen I, as well as p-Smad2 and p-Smad3 protein expressions without affecting the cell viability of TGF-β1 stimulated NRK-49F cells.

**Conclusion:** Gastrodin treatment significantly attenuates hypertensive renal injury and renal fibrosis and suppresses TGF-β1/Smad2/3 signaling *in vivo* and *in vitro*.

## 1 Introduction

Hypertension is a common disease with a high incidence rate and is the most important cause of death due to cardiovascular diseases (Rodriguez-Iturbe et al., 2017). Kidney is not only an important organ causing hypertension but also is the target organ of hypertensive damage (Scheppach et al., 2018). Long-term hypertension leads to various pathological changes in the kidneys, including renal inflammation, renal fibrosis, nephrosclerosis, loss of renal function, and eventually renal failure (Braun et al., 2013; Xue et al., 2016; De et al., 2022). Therefore, reducing renal fibrosis and preventing renal damage is one of the chief strategies for treating hypertension.

Renal fibrosis is a pathophysiological process characterized by tubulointerstitial fibrosis and glomerulosclerosis and is the final outcome of various renal diseases, resulting in chronic organ failure and mortality (Xie et al., 2016; Zhou et al., 2020). During the processes of renal fibrosis, the expressions of mesenchymal marker proteins, such as α-smooth muscle actin (α-SMA) and fibrous matrix proteins (mainly collagen I and III and fibronectin), are significantly upregulated in the renal tissues (Chen et al., 2017; Bülow and Boor, 2019; Schulz et al., 2022). Renal fibrosis is induced and sustained by multiple prosclerotic factors, including transforming growth factor (TGF)-β1, which causes the upregulation of α-SMA, fibronectin, collagen, and other extracellular matrix (ECM) proteins in fibrotic genes and proteins (Jung et al., 2020). TGF-β1 is the best-characterized isoform of the TGF-β superfamily and is a potent fibrogenic cytokine (Jung et al., 2021). TGF-β stimulates ECM accumulation by binding to TGF-β type I and II receptor complexes, which, in turn, activates the TβRI kinases and leads to phosphorylation and activation of Smad2 and Smad3. Once activated, p-Smad2 and p-Smad3 are translocated to the nucleus by forming oligomeric complexes with Smad4 and inducing the expressions of genes responsible for ECM production (Zheng et al., 2018). Therefore, the inhibition of the TGF-β1/Smad pathway might be a viable approach to attenuate hypertensive renal injury and fibrosis.

Gastrodin is a bioactive compound extracted from *Gastrodia elata* (Li et al., 2007). Several studies have reported that gastrodin reduces the blood pressure, protects the heart, delays the formation of atherosclerosis, protects nerve cells, fights central nervous system (CNS) aging, acts as an antiepileptic drug, and alleviates cognitive and emotional disorders (Kung et al., 2021; Liu et al., 2021; Lu et al., 2021; Wang et al., 2021). Gastrodin assuages inflammatory injury of the cardiomyocytes in septic shock mice by inhibiting the expressions of NLRP3 and insulin-like growth factor type 2/insulin-like growth factor type 2 receptor in cardiac hypertrophy and protects the cardiomyocytes from anoxia/reoxygenation injury by the expression of 14-3-3η (Shao et al., 2009; Zhu et al., 2018; Han et al., 2019; Lu et al., 2021). Gastrodin injection alleviates lung injury caused by focal cerebral ischemia in rats *via* the NGF/TrkA pathway-mediated activation of the anti-inflammatory pathway (Chan et al., 2022). Gastrodin protects the CNS and exerts a broad range of beneficial effects on the abovementioned CNS diseases. The mechanisms of action include modulation of neurotransmitters, antioxidative action, anti-inflammatory action, suppression of microglial activation, regulation of mitochondrial cascades, and upregulation of neurotrophins (Liu et al., 2018). Moreover, a previous clinical study has confirmed that gastrodin exerts a significant antihypertensive effect. Gastrodin injection is also widely used in the clinical treatment of patients with hypertension in China (Zhang et al., 2008).

However, the active potential targets and pharmacological mechanisms of gastrodin associated with its antihypertensive effects are still poorly known. Recently, network pharmacology analysis is more effective for establishing a “compound-protein/gene-disease” network and revealing the regulation principles of small molecules in a high-throughput manner (Zhang et al., 2019), which was widely used to explore the pharmacology of a drug and its effect on biological networks and phenotype, as well as its toxicity (Hopkins, 2008). Therefore, potential targets and molecular mechanisms of gastrodin against hypertensive renal injury were explored and verified *via* the *in vivo* and *in vitro* experiments.

## 2 Materials and methods

### 2.1 Materials

Gastrodin (Cat no. B21243) was purchased from Shanghai Yuanye Bio-Technology Co., Ltd (Shanghai, China). Human TGF-β1 (Cat no. AF-100-21C) was obtained from PEPROTECH (Rocky Hill, NJ, United States). Minimum Essential Medium (MEM) Alpha Medium was purchased from Corning (Canton, NY, United States). Fetal bovine serum (FBS), trypsin-EDTA, and bicinchoninic acid (BCA) protein assay reagent kit were purchased from Thermo Fisher Scientific (Waltham, MA, United States). Antigen repair solution (Cat no. MVS-0066) and UltraSensitive™ SP (mouse/rabbit) immunohistochemistry (IHC) (Cat no. KIT-9720) and DAB (Cat no. DAB-0031) kits were purchased from Maixin Biotechnology (Fuzhou, Fujian, China). Masson (Cat no. G1340) and Sirius red (Cat no. PH1099) staining kits were purchased from Solarbio Science and Technology (Beijing, China). Polyvinylidene fluoride (PVDF) membranes were obtained from Millipore (Billerica, MA, United States). Antibodies against α-SMA (Cat no. 19245S) and p-Smad2 (p-Smad2; Cat no.18338S) were purchased from Cell Signaling Technology (Danvers, MA, United States). Antibodies against α-SMA (Cat no. 40482), p-Smad2 (Cat no. 13429), p-Smad3 (Cat no. 12838), Smad2(Cat no. 41442), Smad3(Cat no. 41445) and anti-mouse (Cat no. L3032) and anti-rabbit (Cat no. L3012) secondary antibodies were purchased from Signalway Antibody (College Park, MD, United States). Antibodies against collagen I (Cat no. 14695-1-AP), collagen III (Cat no. 22734-1-AP), and fibronectin (Cat no. 15613-1-AP) were purchased from Proteintech (Rosemont, IL, United States). Antibodies against TGF-β1 (Cat no. ABP52598) and cell counting kit-8 (Cat no. ktc011001) were obtained from Abbkine Scientific (Wuhan, Hubei, China). Antibodies against p-Smad3 (p-Smad3; Cat no. ab52903) and goat polyclonal secondary antibody to rabbit IgG-H&L (Alexa Fluor^®^ 647, Cat no. ab150079) were purchased from Abcam (Cambridge Science Park, Cambridge, UK). Primary and secondary antibody dilution buffers were supplied by Beyotime Biotechnology (Shanghai, China).

### 2.2 Preparation of gastrodin

For animal studies, gastrodin was dissolved in double-distilled water (ddH_2_O) to obtain a dose of 3.5 mg/kg/day based on the average body weight of rats. The dose were selected based on unpublished preliminary study in mice (5 mg/kg/day) adjusting for body surface area and calculating by the following formula: The dose for rat = the dose for mouse (5 mg/kg/day)*body weight of mouse (0.02 kg)* conversion factor (7)/body weight of rat (0.2 kg) = 3.5 mg/kg/day. For cell experiments, the stock solution of gastrodin (1 M) was prepared with dd H_2_O with completely medium to indicate concentrations before use.

### 2.3 Animals and experimental protocols

Ten 4-week-old female spontaneously hypertensive rats (SHRs) and five female Wistar Kyoto (WKY) rats were purchased from Beijing Vital River Laboratory Animal Technology Co., Ltd (Beijing, China). After 2–3 days of adaptive feeding before the experiment, SHRs were randomly categorized into the model (SHR) and treatment (SHR + Gastrodin) groups, with five rats in each group. The five WKY rats served as the control group (WKY). The SHR + Gastrodin group was intragastrically administered with gastrodin daily (3.5 mg/kg/day; 1 ml for each rat), whereas both the WKY and SHR groups were intragastrically administered with equal volumes of dd H_2_O for 10 weeks. All animals were maintained under specific pathogen-free conditions at a constant temperature of 24°C ± 2°C and relative humidity of 50%–60% with a 12-h light–dark cycle. Water and food were provided and were freely accessible to the animals throughout the experiment. The animal experimental protocols were approved by the Animal Care and Use Committee of Fujian University of Traditional Chinese Medicine. The procedures were performed in strict accordance with local and international codes of ethics.

### 2.4 Hematoxylin and eosin (H&E) staining

At the end of experiment, tissues were collected after 10 weeks of gastrodin treatment. Then, renal tissue samples were fixed with 4% paraformaldehyde solution for 48 h, dehydrated, and embedded in paraffin. Subsequently, the tissues were cut into 4-µm sections and stained with hematoxylin for 50 s and eosin for 2 s (H&E staining) to visualize the pathological changes in the renal tissues. The slides were observed under a light microscope (Leica, Wetzlar, Germany) at ×400 magnification. The semi-quantitative percentage of the damaged area was scored as follows: 0, none; 0.5, <10%; 1, 10%–25%; 2, 25%–50%; 3, 50%–70%; and 4, >75%.

### 2.5 Masson’s trichrome staining

Renal tissues were harvested, fixed with 4% paraformaldehyde for 48 h, and embedded in paraffin. The tissues were then sliced into sections of 4-μm thickness for Masson’s trichrome staining. Renal tissue sections were successively immersed in Masson complex for 5 min, acidified with ethanol differentiation solution for 1 s, Masson blue solution for 1 min, Ponceau S red magenta solution for 5 min, distilled water for 1 min, phosphomolybdic acid solution for 1 s, and aniline blue solution for 50 s. The blue pixel content of the images was photographed to quantify renal fibrosis. Five different views were selected under a light microscope in each slide to estimate the values of the integral optical density and total area using the ImageJ software (open source Java image processing program available at https://imagej.nih.gov/ij/). Later, the collagen content was calculated as the percentage of positive area relative to the total area.

### 2.6 Sirius red staining

The renal tissues were sectioned at 6 μm and conventionally dewaxed in water and then were stained with Sirius red staining drops for 1 h, rinsed with running water, then stained with Mayer hematoxylin for 8 min, and rinsed with water for 10 min. The slides were viewed under a Leica DM2700P polarizing microscope (Leica, Wetzlar, Germany) at ×400 magnification. For each rat, the area with collagen fibers was assessed under five microscope fields using Image-Pro-Plus software (Media Cybernetics, Rockville, MD, United States).

### 2.7 Immunohistochemical analysis

The renal tissues were dewaxed and then subjected to heat-induced antigen retrieval in citrate buffer (pH 6.0). The tissues were incubated with the following primary antibodies: α-SMA (1:1,000), collagen I (1:600), collagen III (1:600), TGF-β1 (1:200), p-Smad2 (Cat no. 13429; 1:100), p-Smad3 (Cat no. 12838; 1:75), Smad2(Cat no. 41442; 1:100) or Smad3(Cat no. 41445; 1:100). After overnight incubation at 4°C, the slides were washed with phosphate-buffered saline (PBS) and later incubated with biotinylated secondary sheep anti-mouse/rabbit IgG followed by conjugated horseradish peroxidase-labeled streptavidin. Subsequently, the slides were incubated with diaminobenzidine as the chromogen and counterstained with diluted Harris hematoxylin. The slides were visualized under a Leica DM4000B intelligent automated optical microscope (Leica) and a light microscope at ×400 magnification. Five fields of view were randomly selected for each slide, and the average percentage of positively stained cells in each field was counted using ImageJ software.

### 2.8 Retrieval of gastrodin and hypertensive renal injury targets

The GeneCards was used to retrieve the targets using the term, hypertensive renal injury. Further, DisGeNET was employed for the extraction of targets *via* “renal fibrosis (RF), kidney/renal injury (KI) terms”. Gastrodin targets were collected using pharmamapper and Swiss databases.

### 2.9 Construction and analysis Protein-protein interactions

A search tool for the retrieval of interacting genes (STRING) (https://cn.string-db.org/, Version 11.5) was used to obtain the Protein-protein interaction (PPI) network data of gastrodin and renal fibrosis-related common targets. The interaction network of “gastrodin-renal fibrosis-target” was constructed by using the software of Cytoscape (Version 3.8.2).

### 2.10 Functional annotation and pathways enrichment analysis

Database for annotation, visualization and integration discovery (DAVID) database was employed for the enrichment of gastrodin and renal fibrosis-associated common targets into multiple functional processes and KEGG pathways.

### 2.11 Molecular docking

The most enriched fibrosis-associated targets (TGF-β1 and SMAD2) were selected for molecular docking with gastrodin. The 3D protein structures of TGF-β1 (PDB: 5VQP) and SMAD2 (PDB:1KHX) were retrieved from the protein databank (PDB). Moreover, pocket sites of these proteins were identified by implementing Prankweb online server. Then, the energy of protein structures was minimized by using the dock prep program. Gastrodin and protein structures were optimized and converted into PDBQT format for docking purposes. Furthermore, PyRx software was used for docking by choosing proposed active or pocket sites of the proteins, and proteins-gastrodin docking interactions were visualized by discovery studio.

### 2.12 Cell culture and treatment

The rat kidney fibroblast cell lines (NRK-49F) were obtained from the BeNa (Beijing, China). Cells were cultured in MEM supplemented with 10% FBS and 1% penicillin/streptomycin (100 IU/mL and 100 μg/mL) at 37°C under a 5% CO_2_ atmosphere. The NRK-49F cells were incubated in a serum-free MEM for 4 h and then categorized into the control, TGF-β1, and TGF-β1 + Gastrodin (25, 50, or 100 μM) groups. The cells in the TGF-β1 and TGF-β1+ Gastrodin groups were stimulated with TGF-β1 (5 ng/mL), and those in the TGF-β1 + Gastrodin groups were treated with gastrodin (25, 50, or 100 μM). The cells in the control and TGF-β1 groups were treated with an equal volume of dd H_2_O for a total of 24 h.

### 2.13 Cell viability assay

Cell viability was assessed using the CCK-8 assay according to the manufacturer’s instructions. Briefly, 3 × 10^3^ cells were seeded into each well of 96-well plates for 24 h. Later, the cells were stimulated with TGF-β1 and treated with or without gastrodin (25, 50, or 100 μM) treatment for 24 h. At the end of the experiment, 10 µL of CCK-8 solution was added to each well, and the absorbance was measured at 450 nm using a microplate reader (Multiskan FC, Thermo Fisher Scientific) after 2 h of incubation in the dark. The viability of the untreated cells was taken as 100%.

### 2.14 Immunofluorescence staining

The NRK-49F cells were treated with TGF-β1 (5 ng/mL) alone or TGF-β1 in combination with gastrodin (25, 50, or 100 μM) for 24 h. The cells were fixed with 4% paraformaldehyde for 30 min, followed by permeabilization with 0.25% Triton X-100 in PBS for 5 min at room temperature. After blocking with 10% goat serum for 60 min, the slides were immunostained with anti-fibronectin (1:200), anti-α-SMA (1:200), anti-p-Smad2 (Cat no.18338S; 1:600), or anti-p-Smad3 (Cat no. ab52903; 1:200). The cells were then washed thrice with PBS and incubated with goat polyclonal secondary antibody to rabbit IgG-H&L (diluted 1:200) for 1 h. After washing with PBS, the cells were stained with hoechst for 10 min and then washed twice with PBS. Finally, the stained cells were visualized under a confocal microscope (UltraVIEW ®Vox, PerkinElmer, Santa Clara, CA, United States ). The images were acquired at ×600 magnification. The fluorescence intensity of the stained cells was calculated using Volocity software (PerkinElmer).

### 2.15 Western blotting

The NRK-49F cells were cultured in 6-well plates to a density of 0.8 × 10^5^ cells per well. After being cultured for 24 h, the NRK-49F cells were incubated in a blank medium for approximately 6 h. Then, the cells were stimulated with TGF-β1 and treated with or without gastrodin (25, 50, or 100 μM) for 48 h. Cell lysis buffer containing protease inhibitor and PMSF was added to the renal tissues, which were then ground using a low-temperature grinder, kept on ice for 30 min, and centrifuged at 4°C and 14,000 g for 20 min. The protein concentration was estimated using the BCA assay. A total of 50 μg of protein from each sample was separated on 10% SDS-PAGE gel, transferred to PVDF membranes, and blocked with 0.5% bovine serum albumin for 2 h. This step was followed by incubation with the primary antibodies (overnight at 4°C), including rabbit anti-collagen I (1:1,000), mouse anti-α-SMA (1:10,000), rabbit anti-fibronectin (1:1,000), and rabbit anti-glyceraldehyde-3-phosphate dehydrogenase (GAPDH) (1:5,000). Subsequently, the membrane was washed with TBST and then incubated with anti-mouse (1:10,000) and antirabbit (1:5,000) secondary antibodies at room temperature for 1 h. At the end of incubation, the membranes were washed with TBST and detected using an electrochemiluminescence kit. The expression of GAPDH protein was used as an internal control. The protein levels were analyzed with ImageJ software. The gray value of each protein band was normalized to that of the corresponding GAPDH band. The expressions of these proteins were normalized to those of the control group, which was set as 1.

### 2.16 Statistical analysis

Statistical analysis was performed using the SPSS statistical program (SPSS/PC+, version 22.0, Chicago, IL, United States). The results were presented as the mean value ±standard deviation (SD). One-way analysis of variance was used to compare the differences among three or more groups when the data were normally distributed. Then the LSD was used when the variance was chi-square, and the Games Howell was used when the variance was not chi-square. The non-parametric Kruskal–Wallis test was used to compare the differences among three or more groups when the data were not normally distributed, selected Pairwise Comparisons in the view option. Differences associated with *p* < 0.05 were considered statistically significant.

## 3 Results

### 3.1 Gastrodin ameliorated hypertensive renal injury and attenuated renal interstitial fibrosis in SHRs

**FIGURE 1:**
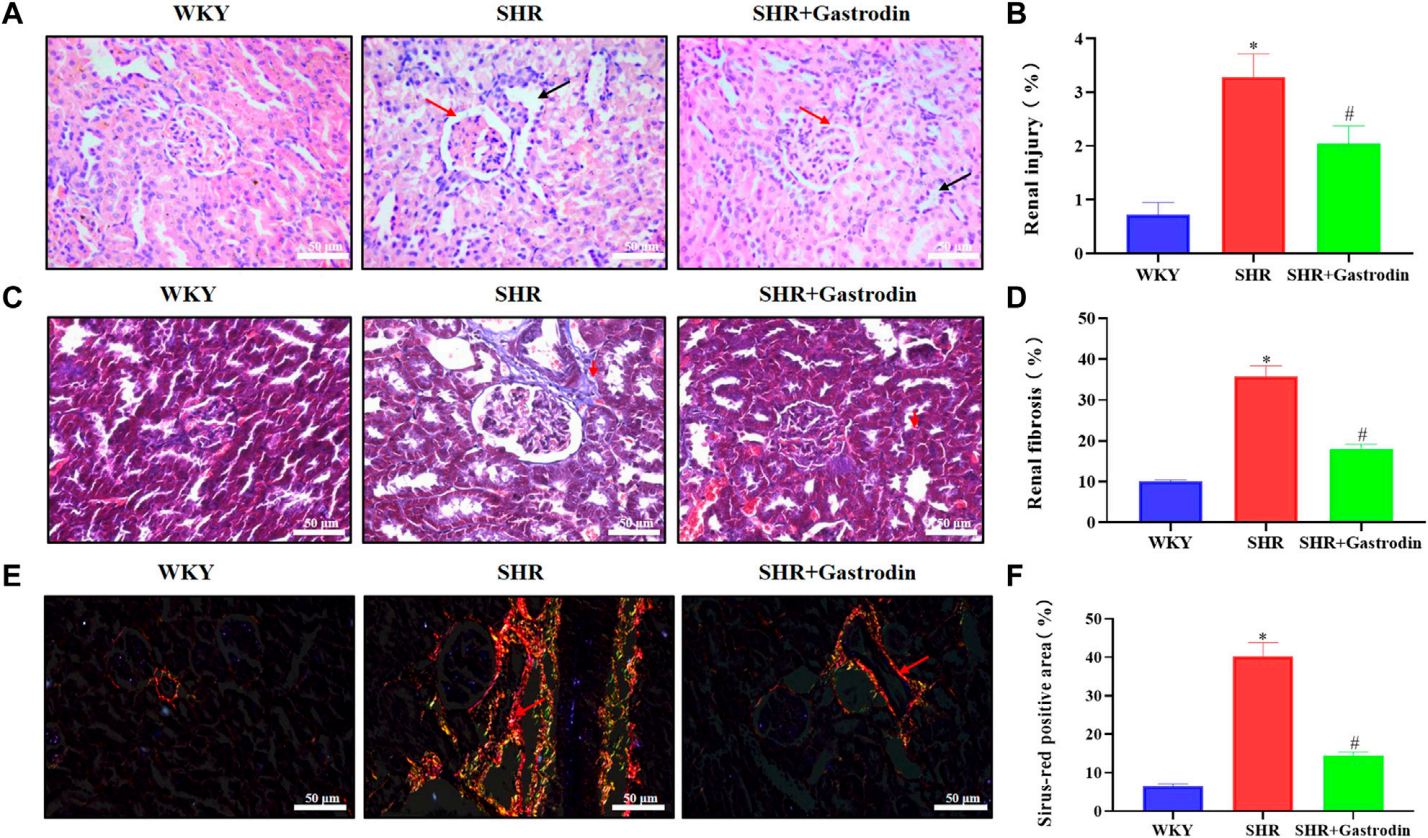
Gastrodin ameliorated hypertensive renal injury and renal interstitial fibrosis in spontaneously hypertensive rats (SHRs) **(A,B)** Hematoxylin and eosin (H&E) staining was performed to determine the pathological changes in the renal tissues from each group. The representative images were taken at ×400 magnification (scale bar 50 μm). *N* = 5 for each group **(C,D)** Masson’s trichrome staining was performed to assess the degree of renal fibrosis in each group **(E,F)** Sirius red staining was performed to detect the accumulation of collagen fibers in the renal tissue. All micrographs were taken at ×400 magnification. Scale bar = 50 μm. All values are mean ± standard deviation (SD) (*n* = 5 for each group). *p < 0.05 vs the WKY group, #*p* < 0.05 vs the SHR group.

H&E staining revealed histopathological changes in the renal cortex after gastrodin treatment (Figures 1A, B). Compared with the WKY kidneys, the SHR kidneys exhibited obvious contraction and thickening of the glomerular atrophy, glomerular basement membrane (red arrow), and dilated tubules (black arrow). Gastrodin significantly alleviated hypertension-associated glomerular contraction and basement membrane thickening in the lesion area of SHR kidneys. The degree of collagen fiber formation in the renal tissues of SHRs was measured using Masson’s trichrome staining and Sirius red staining. Masson’s staining revealed that collagen fiber deposition in the interstitial space of the renal tubule was more extensive in the SHR group than that in the WKY group (Figures 1C, D; *p < 0.05 vs the WKY group), which was attenuated after gastrodin treatment (Figures 1C, D; #*p* < 0.05, vs the SHR group). The Sirius red staining images showed that the collagen fibers in the renal tissues were significantly higher in the SHR group than those in the WKY group (Figures 1E, F; *p < 0.05 vs the WKY group), although this was reduced after gastrodin treatment (Figures 1E, F; #*p* < 0.05, vs the SHR group).

### 3.2 Gastrodin reduced the expressions of fibrosis markers in SHRs

**FIGURE 2:**
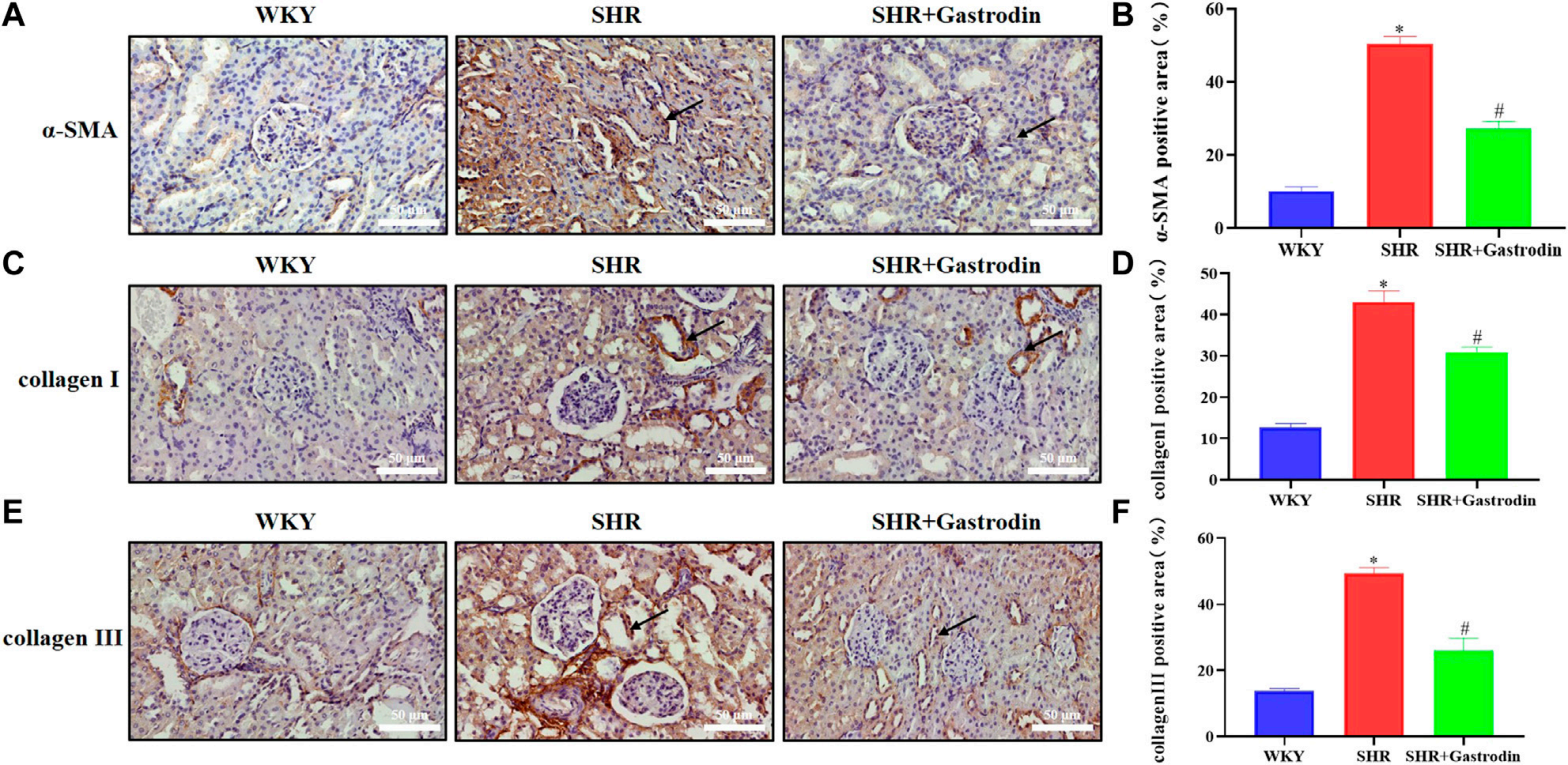
Gastrodin reduced the expressions of fibrosis markers in spontaneously hypertensive rats (SHRs) **(A)** Immunohistochemistry (IHC) was performed to measure the levels of α-smooth muscle actin (α-SMA) in the renal tissues of rats. The positive area in **(B)** α-SMA staining cells in rats was calculated. IHC was performed to detect the expressions of **(C)** collagen I and the positive area in **(D)** collagen I staining cells in rats was calculated in the renal tissues. IHC was performed to detect the expressions of **(E)** collagen III and the positive area in **(F)** collagen III staining cells in rats was calculated in the renal tissues.All micrographs were taken at ×400 magnification. Scale bar = 50 μm. All values are mean ± SD (*n* = 5 for each group). *p < 0.05 vs the WKY group, #*p* < 0.05 vs the SHR group.

We explored the potential effects of gastrodin on renal injury by analyzing the expression of α-SMA using IHC staining of the renal tissue. The results revealed that the renal tissue of SHRs showed significant upregulation of α-SMA (Figures 2A, B; *p < 0.05 vs the WKY group), which was reversed after gastrodin treatment (Figures 2A, B; #*p* < 0.05 vs the SHR group). To further examine the type of collagen fiber formation, we used IHC staining to analyze the expressions of collagen I and collagen III in the renal tubule interstitium. SHRs demonstrated significant upregulation of collagen I and collagen III (Figures 2C–F; *p < 0.05 vs the WKY group), which was reversed after gastrodin treatment (Figures 2C–F; #*p* < 0.05 vs the SHR group). Thus, gastrodin reduced collagen deposition in the renal tissues of SHRs.

### 3.3 Network of Protein-Protein Interactions (PPI) of common targets between gastrodin and renal fibrosis targets

**FIGURE 3:**
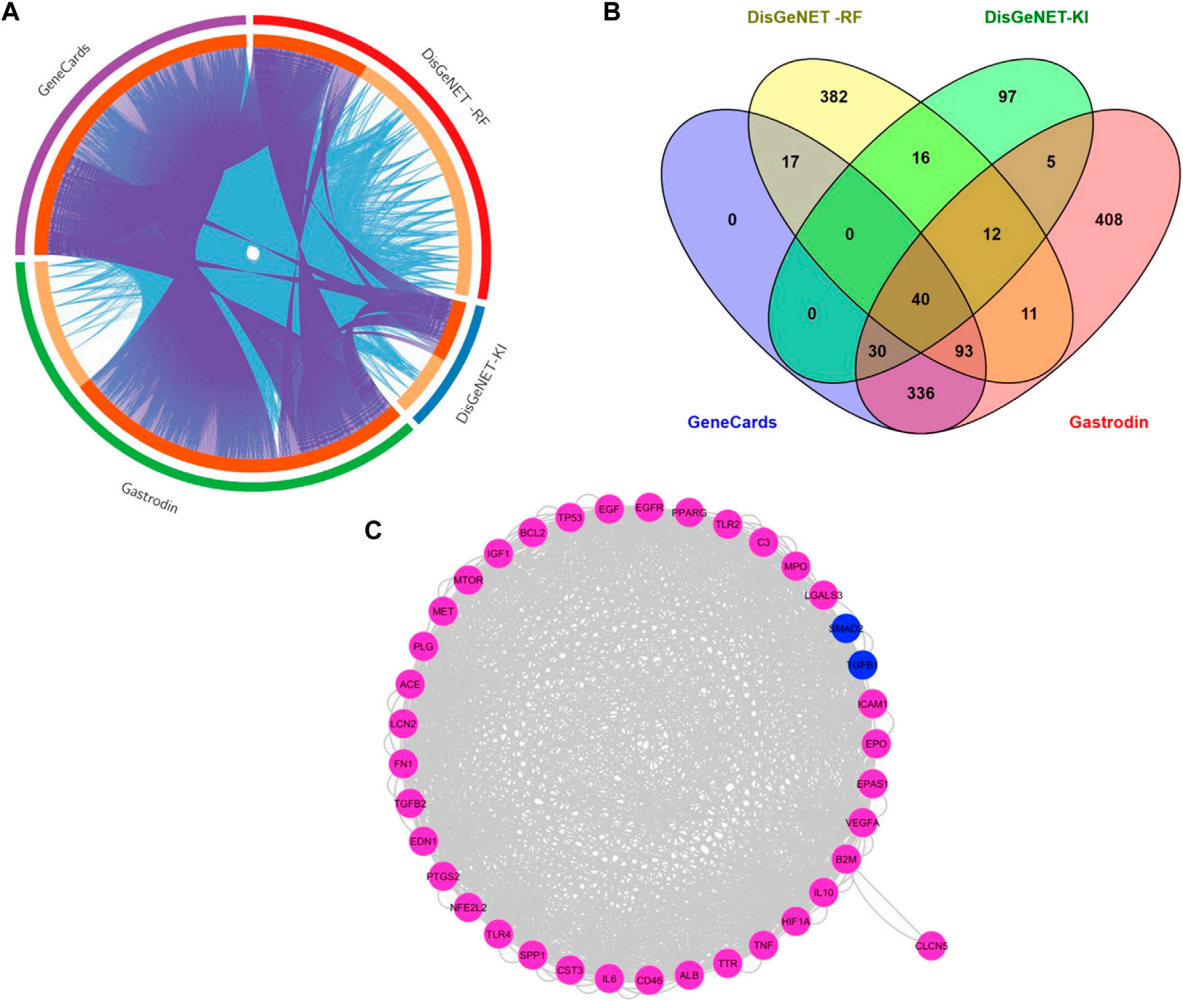
Network of Protein-Protein Interactions (PPI) of common targets between gastrodin and renal fibrosis targets **(A)**The Circos plot shows intersecting genes. The outer arcs symbolize the identity of each gene. Each of the inside arcs represents a gene, and each gene is represented by its own spot on the arc. The dark orange color indicates genes found in multiple databases, whereas the light orange color signifies genes found only in that gene datasets. Purple lines connect identical gene that seems in specific genes databases. Blue lines connect the genes that are identified as similar ontologies **(B)** Overlapped targets between gastrodin and renal fibrosis **(C)** Network construction of common genes.

The intersection of targets from different databases is represented into Circos diagram. Gene searches were performed using these terms, renal fibrosis (RF), renal/renal injury (KI), hypertensive renal injury and gastrodin using pharmamapper and stitch databases, as well as the Swiss database (Figure 3A). The overlapping gene analysis was performed and 40 common genes were found (Figure 3B). We entered these 40 common targets in the STRING database and obtained the network relationship data of target interaction under the threshold of a minimum required interaction score >0.4. Each edge represents the interaction between protein and protein (Figure 3C). The list of common 40 targets is given in the Supplementary Table S1.

### 3.4 Evaluation of Gene Ontology and Pathways

**FIGURE 4:**
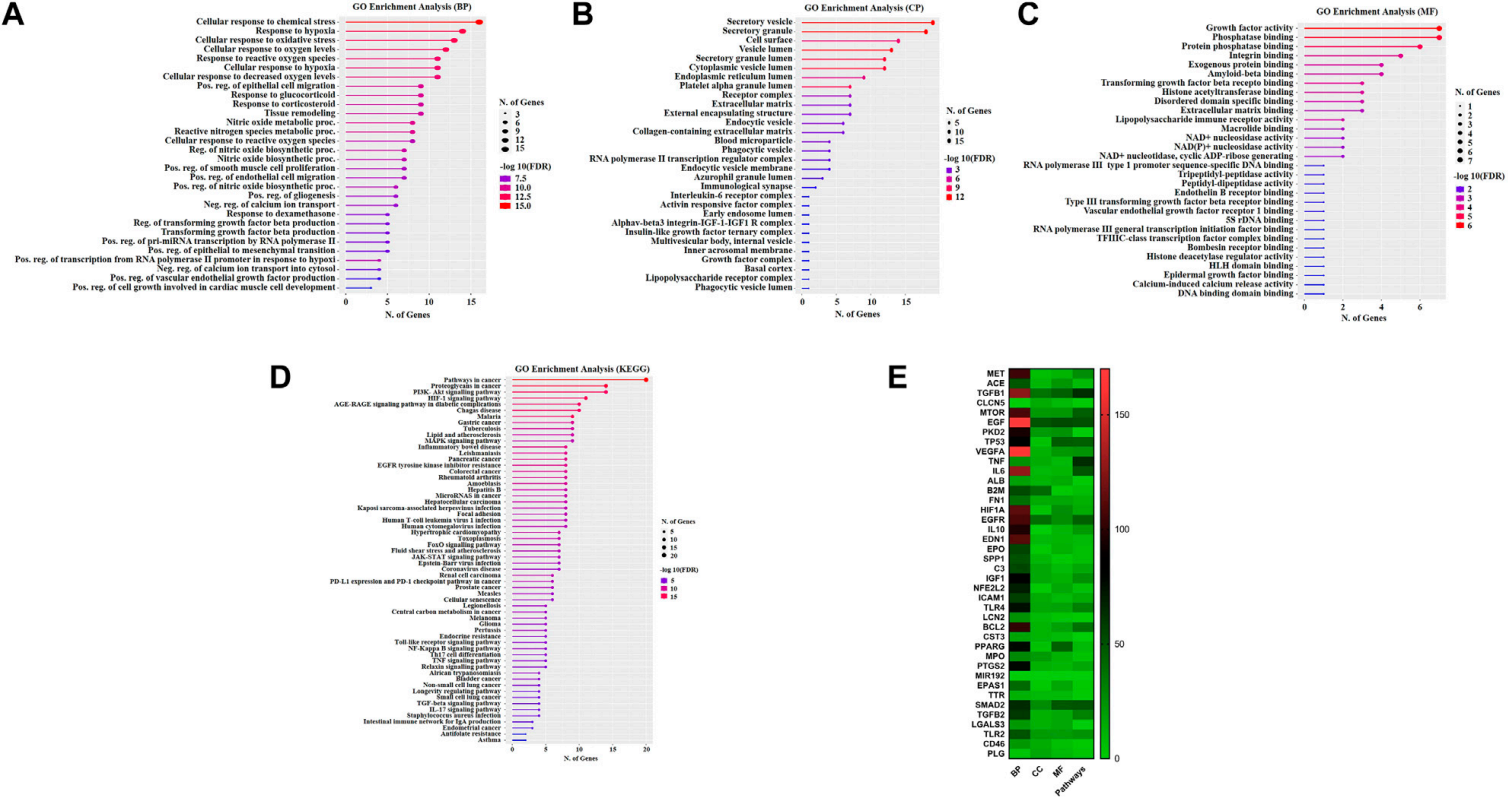
Evaluation of Gene Ontology and Pathways. GO and KEGG pathways enrichment analysis with Gastrodin. The *y*-axis depicts terms that are greatly enriched, while the number of genes of these terms is shown on the *x*-axis **(A)** Biological processes (BP) **(B)** Cellular component (CC) **(C)** Molecular function (MF), and **(D)** KEGG pathways categories **(E)** Quantitative comparison analysis of all 40 proteins into GO terms and pathways. The *y*-axis of the heatmap shows the name of proteins, the *x*-axis GO terms biological processes (BP), cellular component (CC), and molecular function (MF), and pathways.

The GO analysis was performed using DAVID database to clarify the mechanism of gastrodin’s behavior on hypertensive renal injury. After entering these 40 common targets were significantly enriched into several biological processes, cellular processes, and molecular functions using (FDR cutoff 0.05) and were categorized separately. The top 40 processes of each GO term were represented in figures and details were given in Supplementary Table S2. In hypertension pathogenesis, these targets were involved in vasculature development, pos. reg. of cell population proliferation and wound healing, reg. of epithelial cell proliferation, epithelial cell proliferation, and blood vessel development. TGF-β1 is involved in the formation of these biological processes (Figure 4A). The main cellular component also consisted of secretory granules, vesicle lumen, and secretory vesicle (Figure 4B). In addition, the category of molecular functions included signaling receptor binding, receptor-ligand activity, signaling receptor activator activity, and growth factor activity (Figure 4C). KEGG pathway enrichment analysis by the DAVID database using FDR cutoff 0.05 and identified multiple signaling pathways MAPK signaling pathway and FoxO signaling pathway, notably multiple inflammation or oxidative stress associated pathways, including TNF signaling pathway, Th17 cell differentiation, Toll like receptor, NF-kappa B signaling pathways, and so on (Figure 4D; Supplementary Table S2). Furthermore, quantitative comparison of all 40 proteins in GO terms and pathways is shown (Figure 4E). TGF-β1 and Smad2 proteins were abundantly enriched into 126 and 65 biological processes out of 371 processes respectively. In pathways analysis, TGF-β1 and Smad2 were quantitatively enriched into 64 and 49 pathways out of 135 pathways respectively.

### 3.5 Evaluation of molecular docking studies

**FIGURE 5:**
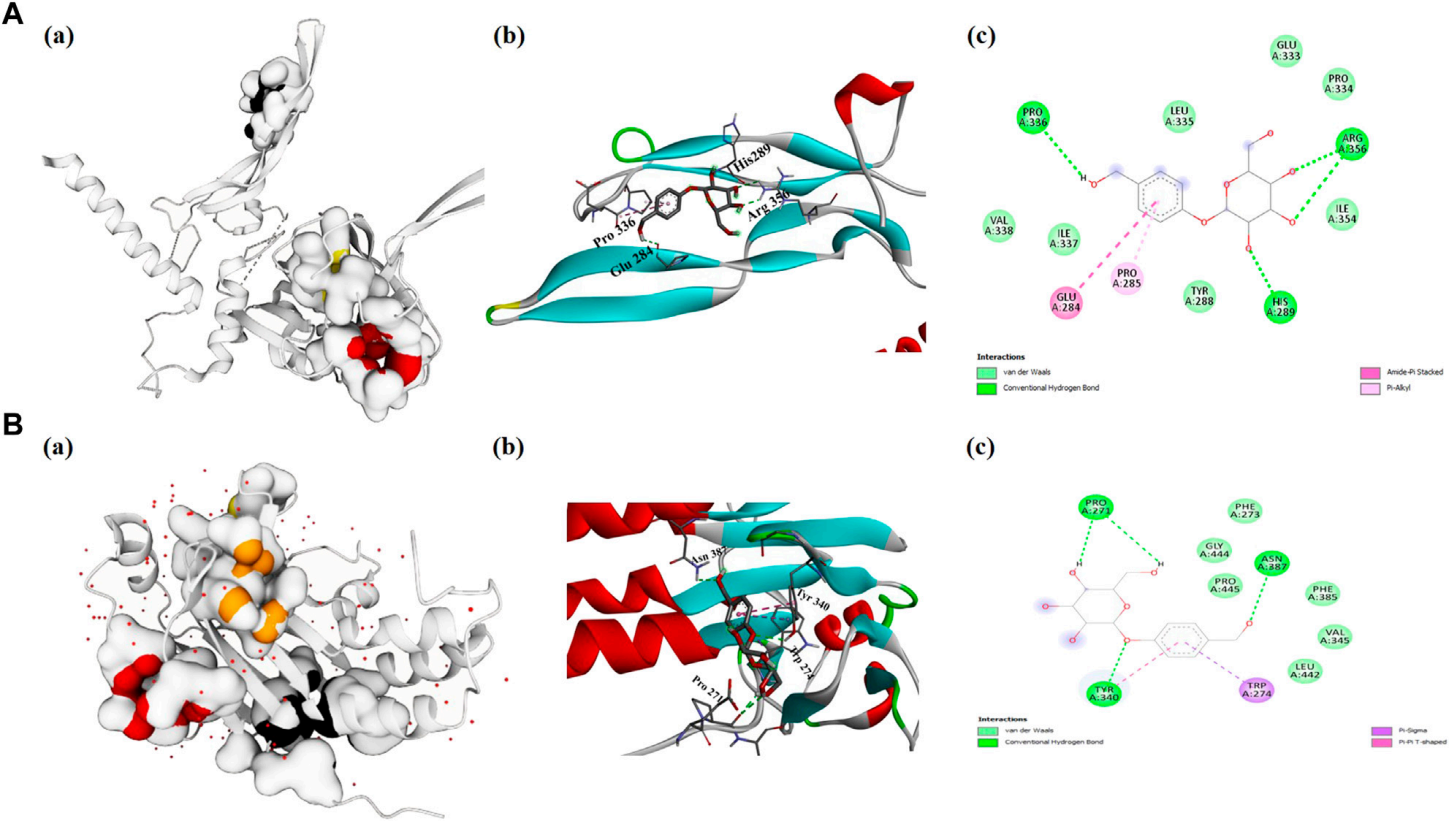
Evaluation of molecular docking studies. Representation of protein-ligand complex interaction **(A)** TGF-β1 **(A)** shows the prediction of pocket sites **(B)** docking of gastrodin-TGF-β1 receptor and **(C)** 2D-interacting residues of TGF-β1 to gastrodin molecule **(B)** SMAD2 **(A)** represents the pocket sites **(B)** docked representation of gastrodin-SMAD2 receptor and **(C)** 2D-interaction of SMAD2 amino acids with gastrodin.

The TGF-β1 and SAMD2 proteins were found abundantly enriched into most GO categories and pathways. Therefore, these targets were considered renal fibrosis potential targets and docked with gastrodin in order to provide a deeper insight into the binding interactions of the inhibitor (gastrodin) at the active sites. Docking energies and related docking residue sites are shown in Table 1. It is generally believed that the lower the docking energy between ligands and proteins, the stronger their bond. The results analysis showed that TGF-β1-gastrodin had a high binding affinity −5.1 kcal/mol (Figure 5A). Furthermore, best docking binding energy −6.6 kcal/mol was determined between SMAD2 and gastrodin (Figure 5B).Thus, this molecular docking results suggest that gastrodin might be one of the potential inhibitor molecule for renal fibrosis.

**TABLE 1:** Docking energies and bonds between gastrodin and proteins.

Compound name	Proteins	Docking energies (kcal/mol)	Functional groups	Protein residues	Bonds
Gastrodin	TGF-β1 (PDB: 5VQP)	−5.1 kcal/mol	H	Pro 336	H-bond
			O	Arg 356	H-bond
			O	His 289	H-bond
Gastrodin	SMAD2 (PDB:1KHX)	−6.6 kcal/mol	O	Asn 387	H-bond
			O	Pro 271	H-bond
			H	Tyr 340	H-bond

### 3.6 Gastrodin suppressed the TGF-β1/Smad2/3 signaling pathway *in vivo*

**FIGURE 6:**
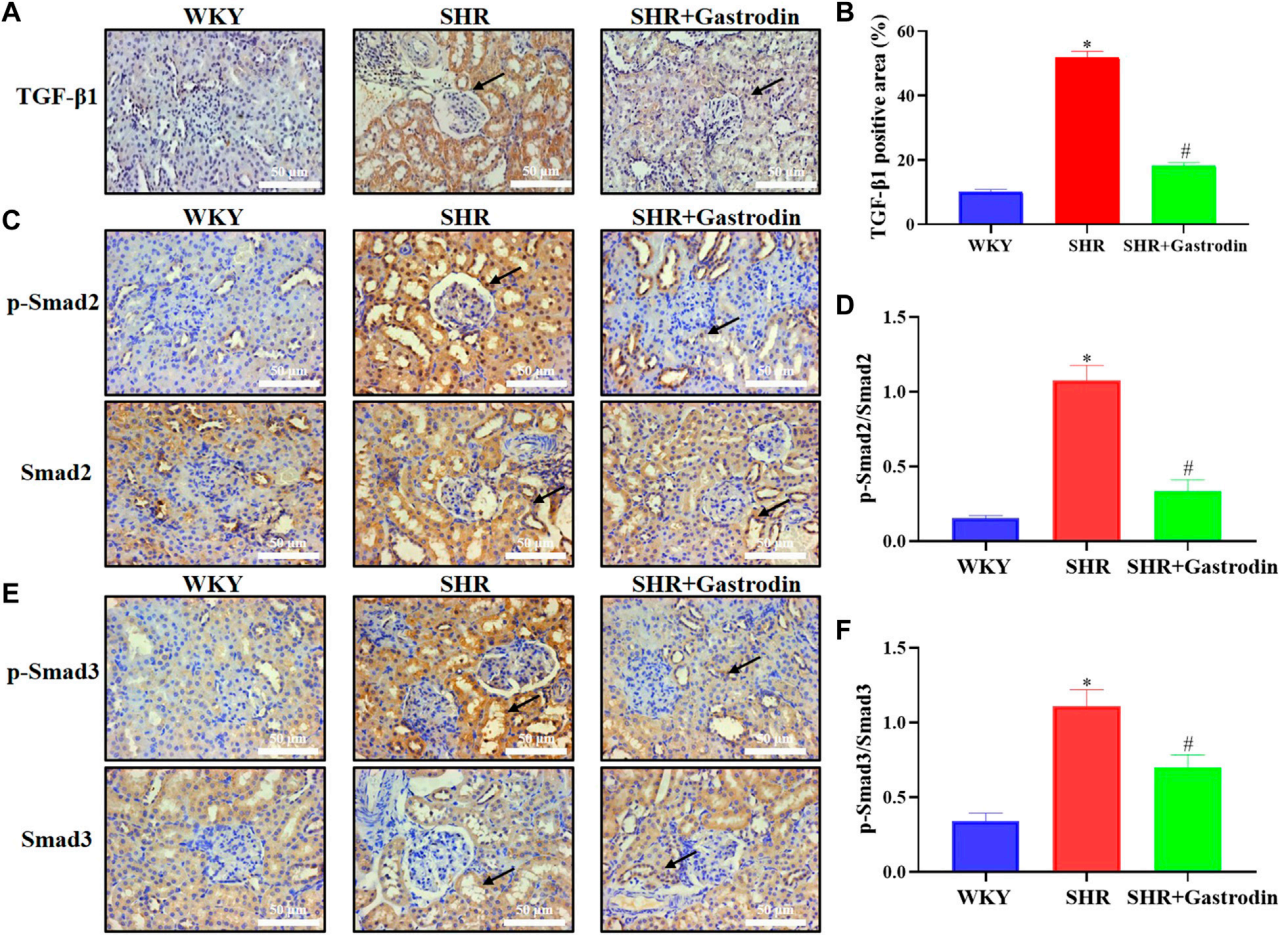
Gastrodin inhibited the TGF-β1/Smad2/3 signaling pathway *in vivo* **(A)** Immunohistochemistry (IHC) was performed to measure the levels of TGF-β1 in the renal tissues of rats. The positive areas in **(B)** TGF-β1 staining cells in rats were calculated. IHC was performed to detect the expressions of **(C)** ratio of p-Smad2/Smad2 protein and the positive areas in **(D)** p-Smad2/Smad2 staining cells in rats were calculated in the renal tissues. IHC was performed to detect the expressions of **(E)** ratio of p-Smad3/Smad3 protein and the positive areas in **(F)** p-Smad3/Smad3 staining cells in rats were calculated in the renal tissues. All micrographs were taken at ×400 magnification. Scale bar = 50 μm. All values are mean ± SD (*n* = 5 for each group). *p < 0.05 vs the WKY group, #*p* < 0.05 vs the SHR group.

Owing to the essential role of the TGF-β/Smad pathway in the development of hypertensive renal fibrosis, we explored the underlying mechanism of the renal protective effect of gastrodin using IHC. The results of IHC staining revealed that the renal tissue of SHRs exhibited a significant increase in TGF-β1, and ratio of p-Smad2/Smad2, p-Smad3/Smad3, which were attenuated after gastrodin treatment (Figures 6A–F; *p < 0.05 vs the WKY group; #*p* < 0.05 vs the SHR group). These results suggested that gastrodin treatment attenuates the activation of the TGF-β1/Smad2/3 signaling pathway in renal tissues of SHRs.

### 3.7 Gastrodin suppressed the expressions of α-SMA and fibronectin in TGF-β1-treated NRK-49F cells

**FIGURE 7:**
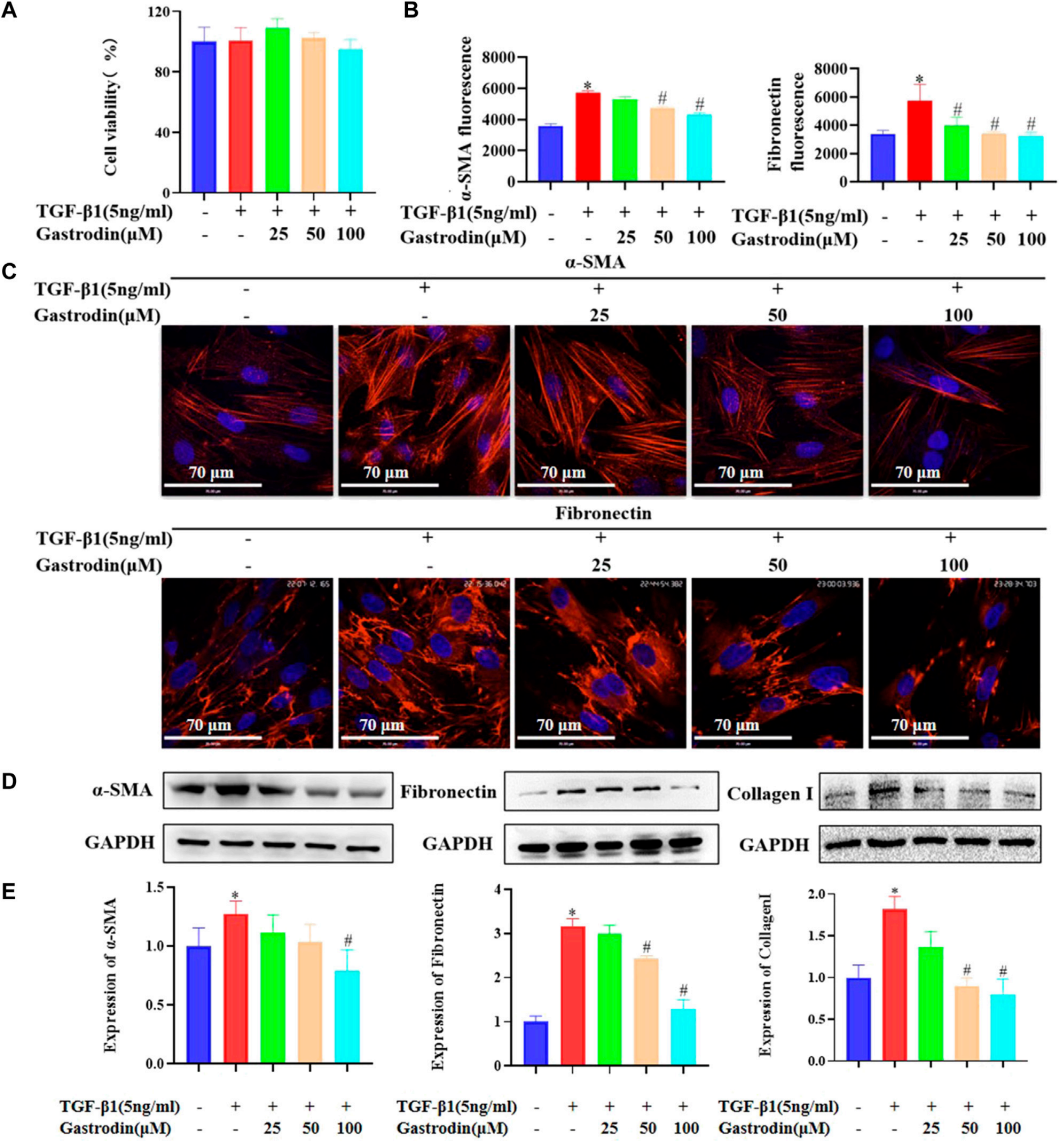
Gastrodin inhibited the expressions of α-smooth muscle actin (α-SMA) and fibronectin in transforming growth factor-β1 (TGF-β1)-treated NRK-49F cells **(A)** Viability of NRK-49F cells after treatment with gastrodin and TGF-β1, as analyzed using the CCK-8 assay. The viability of untreated NRK-49F cells was defined as 100%. Data are mean ± SD (*n* = 6 for each group) **(B)** Relative α-SMA and fibronectin expressions in each group were determined using ImageJ. All values are mean ± SD (*n* = 3 for each group). *p < 0.05 vs the control group, #*p* < 0.05 vs the TGF-β1 group **(C)** Immunofluorescence (IF) staining was performed to detect the expressions of α-SMA and fibronectin (red). The nuclei (blue) were stained with Hoechst. Magnification, ×600 (scale bar 70 μm) **(D)** Western blot analysis was performed to determine the protein expressions of α-SMA, fibronectin, and collagen **(I)**. GAPDH was used as the internal control **(E)** Data were presented as mean ± SD; *p < 0.05 vs the control group, #*p* < 0.05 vs the TGF-β1 group.

We examined the effects of the combination of gastrodin and TGF-β1 on cell viability and noted that 100 μM gastrodin and 5 ng/mL TGF-β1 did not affect the cell viability (Supplementary Figures S1A, B; Figure 7A; *p < 0.05 vs the control group). Therefore, 100 μM gastrodin and 5 ng/mL TGF-β1 were used for the subsequent *in vitro* experiments. Immunofluorescence analysis indicated that gastrodin treatment partially reversed the TGF-β1-induced increase in the expressions of α-SMA and fibronectin (Figures 7B, C; *p < 0.05 vs the control group; #*p* < 0.05 vs the TGF-β1 group) in the NRK-49F cells. Moreover, determination of the expression of the fibrosis-related proteins using western blot analysis revealed that TGF-β1 infusion significantly upregulated the expressions of α-SMA, fibronectin, and collagen I, which were all reversed after gastrodin treatment (Figures 7D, E; *p < 0.05 vs the control group; #*p* < 0.05 vs the TGF-β1 group).

### 3.8 Gastrodin suppressed the TGF-β1/Smad2/3 signaling pathway *in vitro*

**FIGURE 8:**
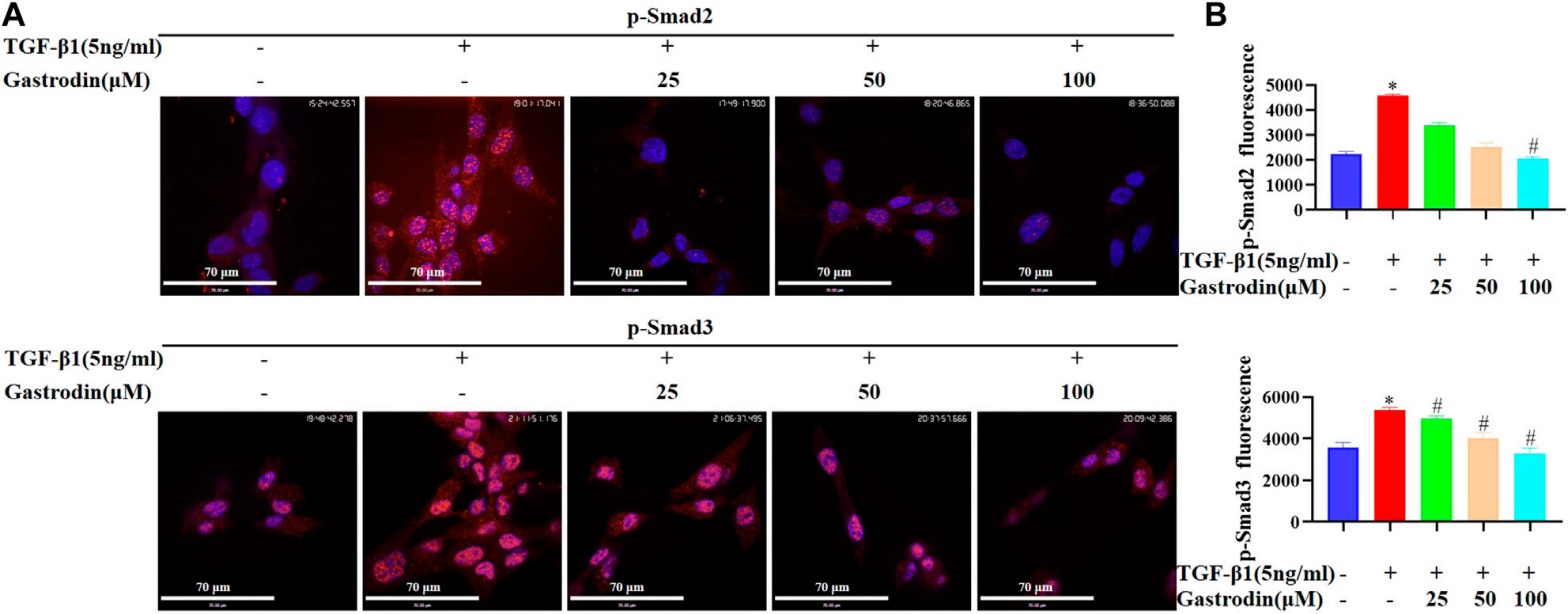
Gastrodin inhibited the TGF-β1/Smad2/3 signaling pathway *in vitro* **(A)** Immunofluorescence (IF) staining was performed to detect the expressions of p-Smad2 (p-Smad2) and p-Smad3 (p-Smad3) (red). Nuclei (blue) were stained with Hoechst. Magnification, ×600 (scale bar 70 μm) **(B)** Relative p-Smad2 and p-Smad3 expressions in each group were determined using ImageJ. All values are mean ± SD (*n* = 3 for each group). *p < 0.05 vs the control group, #*p* < 0.05 vs the TGF-β1 group.

Consistently, TGF-β1 significantly upregulated the p-Smad2 and p-Smad3 (both mainly located in the nucleus) in the NRK-49F cells, which were reduced after gastrodin treatment (Figures 8A, B; *p < 0.05 vs the control group; #*p <* 0.05 vs the TGF-β1 group).

## 4 Discussion

The findings from previous study showed that gastrodin injection combined with conventional therapy can improve systolic blood pressure and diastolic blood pressure, so that can be used as a supplementary treatment for hypertension (Qian et al., 2020). Moreover, pretreatment with gastrodin may alleviate renal ischemia-reperfusion injury *via* the amelioration of oxidative injury, inflammatory response, and renal tubular apoptosis (Zheng et al., 2022). Gastrodin exerts a nephroprotective effect against lead-induced oxidative stress and inflammation in mice *via* the GSH, Trx, and Nrf2 antioxidant system and the HMGB1 pathway (Tian et al., 2021). The effects of gastrodin against carbon tetrachloride-induced kidney inflammation and fibrosis in mice *via* the AMPK/Nrf2/HMGB1 pathway have also been reported (Ma et al., 2020). However, the key molecules involved in the renal protective mechanism have not been completely elucidated.

In this study, we observed that gastrodin alleviated renal injury caused by hypertension in SHRs. In the course of renal injury, excessive accumulation of ECM in the glomerulus and renal interstitium has been shown to be the main pathologic change associated with renal fibrosis (Hosoo et al., 2015; Yang et al., 2020). Renal fibrosis promotes fibroblast activation and cell proliferation and production of α-SMA, collagen I, collagen III, and fibronectin (Yuan et al., 2019). The evaluation of renal damage has been restricted to the renal cortex, and potential underlying alterations in the renal medulla have not been included. In the present study, gastrodin was shown to decrease renal fibrosis and inhibit the expressions of α-SMA, collagen I, and collagen III in the SHR model as well as downregulate α-SMA and fibronectin in the TGF-β1-treated NRK-49F cells. During the period of hypertension, reactive oxygen species and lipid peroxidation products (MDA) increase (Reddy et al., 2009; Senador et al., 2010). Oxidative stress response in the renal cells activates various transcription factors, such as NF-κB, and increases the expression of cytokine TGF-β1 (Seccia et al., 2017; Wang et al., 2018; Zhao et al., 2019; Qiu et al., 2020). In the future, we intend to investigate whether gastrodin can protect against hypertension-induced kidney damage by reducing the inflammatory response and oxidative stress.

Numerous studies have shown that multiple signaling pathways (including PI3K/AKT, STAT3 Notch, Wnt/beta-catenin, and Hedgehog) are associated with renal fibrosis fibers (Yang and Liu, 2001; Iwano et al., 2002; Derynck and Zhang, 2003; Lan, 2003; Wang et al., 2005). In this study, a network pharmacology approach combined with experimental validation was used to study the possible renal injury pharmacological mechanisms of gastrodin. Our results identified the overlapping gene analysis was performed and 40 common genes were found between gastrodin and renal fibrosis targets. In addition, we used network pharmacology analysis and obtained potential gene targets, including TGF-β1 and SMAD2 with different binding potential with gastrodin. KEGG pathway analysis showed several pathways related to renal inflammation, involveding PI3K-AKT signaling pathway, MAPK signaling pathway, JAK-STAT signaling pathway, NF-kappa B signaling pathway, TNF signaling pathway. And the pathway related to renal oxidative stress, involving FoxO signaling pathway. However, TGF-β1 is a key inducer of renal fibrosis, and renal inflammation and oxidative stress are the influencing factors for inducing TGF-β1/Smad2/3 signaling pathway (Yuan et al., 2022). The above studies highlight the potential underlying mechanism of gastrodin against hypertensive renal injury *via* TGF-β1/Smad2/3 signaling pathway by targeting TGF-β1 and SMAD2. TGFβ-Smad2/3 is a key pathway in the regulation of renal fibrosis (Xu et al., 2021; Liu et al., 2022). Molecular docking is the most frequently employed strategy for evaluating protein-ligand interactions, and we employed the PyRx software to scrutinize the probable binding affinity. Our docking analysis showed strong binding affinities between gastrodin, TGF-β1 and SMAD2 complexes. Gastrodin forms three hydrogen bonds with TGF-β1 amino acids residues His 289, Pro336 and Arg 356 and it also form three hydrogens with SMAD2 residues, Pro 271, Tyr 340 and Asn 387. These results showed that gastrodin has a best activity, and it can dock well with renal fibrosis or renal injury targets, which is consistent with the results of a long-term study showing that gastrodin is an effective drug for the treatment of renal injury (Ma et al., 2020).

Moreover, the activation of the TGF-β1 receptor transfers phosphorylated Smad2 and Smad3 with Smad4 in the form of oligomeric complexes to the nucleus and regulates the transcription of target genes. This initiates the transdifferentiation of renal tubular epithelial cells into myofibroblasts and promotes collagen and fibronectin synthesis and ECM deposition (He et al., 2009; Zhou et al., 2014; Yu et al., 2021; Park et al., 2022). Our findings signified that gastrodin intervention in SHRs significantly inhibited the expression of TGF-β1 and decreased the expression of ratio of p-Smad2/Smad2, and p-Smad3/Smad3. Additionally, gastrodin treatment attenuated TGF-β-induced endonuclear phosphorylation of Smad2 and Smad3 in the NRK-49F cells, which correlated with the mitigation of fibrosis. Furthermore, more work is needed to further explore the underlying mechanisms of gastrodin in sequenced potential target genes (TGF-β and SMAD2) and other signaling pathways based on network pharmacology analysis. However, there are limitations in our study, the lack of treatment group in healthy rats (WKY + Gastrodin). In fact, this could well be helpful to dissect pathways affected by gastrodin treatment in the comparison of disease development and healthy animals. And the small sample size of animal experiments did not provide enough data to prove that gastrodin has a good effect on hypertension-induced fibrosis. Moreover, kidney function was not examined in the present study, such as serum levels of Cr and BuN by ELISA. Another issue is no comparison between gastrodin and other components of currently-used treatment of fibrosis or medications for SHR/kidney fibrosis. We will also further explore the regulatory effect of gastrodin on others targets and pathways, as well as related molecular functions in our recently study. Further work should investigate the renal injury due to hypertension, including its potential molecular mechanisms and therapeutic applications.

## 5 Conclusion

This study demonstrated that gastrodin treatment significantly attenuates hypertensive renal injury and renal fibrosis. Gastrodin also suppresses TGF-β1/Smad2/3 signaling *in vivo* and *in vitro*. These results provide experimental evidence for the therapeutic potential of gastrodin against renal injury and renal fibrosis.

## Data Availability

The original contributions presented in the study are included in the article/Supplementary Material, further inquiries can be directed to the corresponding authors.

## Abbreviations

α-SMA, α-smooth muscle actin; BCA, bicinchoninic acid; CCK8, cell counting kit-8; dd H_2_O, double-distilled water; ECM, extracellular matrix; FBS, fetal bovine serum; GAPDH, glyceraldehyde-3-phosphate dehydrogenase; GO, gene ontology; H&E, hematoxylin–eosin; IHC, immunohistochemistry; Kyoto; IRI, ischemia–reperfusion injury; KI, kidney/renal injury; MEM, minimum essential medium; NRK-49F, rat kidney fibroblast cell lines; PBS, phosphate-buffered saline; PDB, protein databank; PPI, Protein-Protein Interactions; PVDF, polyvinylidene fluoride; RF, renal fibrosis; SHRs, spontaneously hypertensive rats; TGF-β, transforming growth factor-β; WKY, Wistar.
