# Supplementary material for: Gastrodin attenuates renal injury and collagen deposition via suppression of the TGF-β1/Smad2/3 signaling pathway based on network pharmacology analysis
Source: Front Pharmacol. 2023 Jan 17;14:1082281. doi: 10.3389/fphar.2023.1082281 (PMC9887022; doi:10.3389/fphar.2023.1082281)

Figure1 A

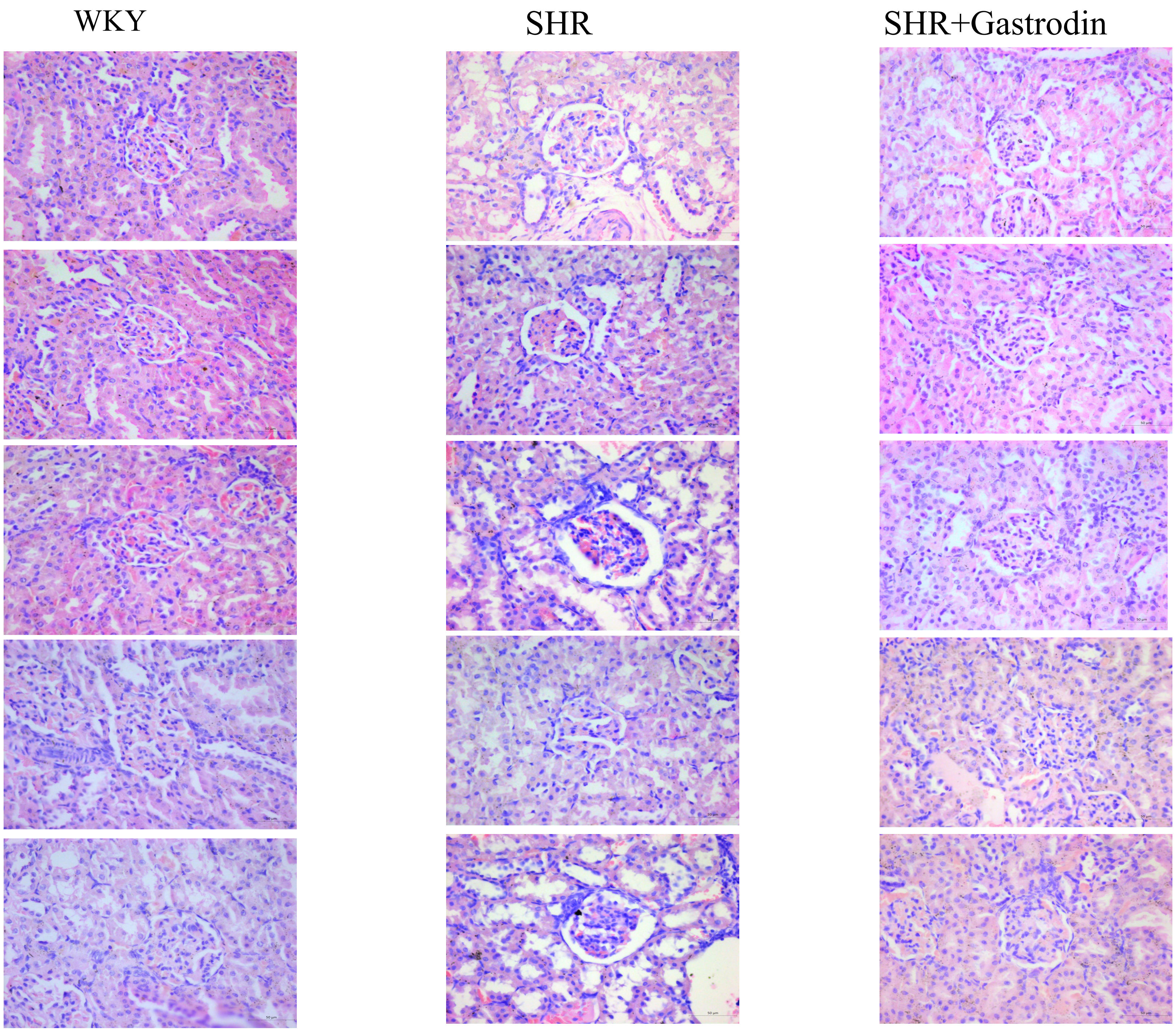

Figure1 C

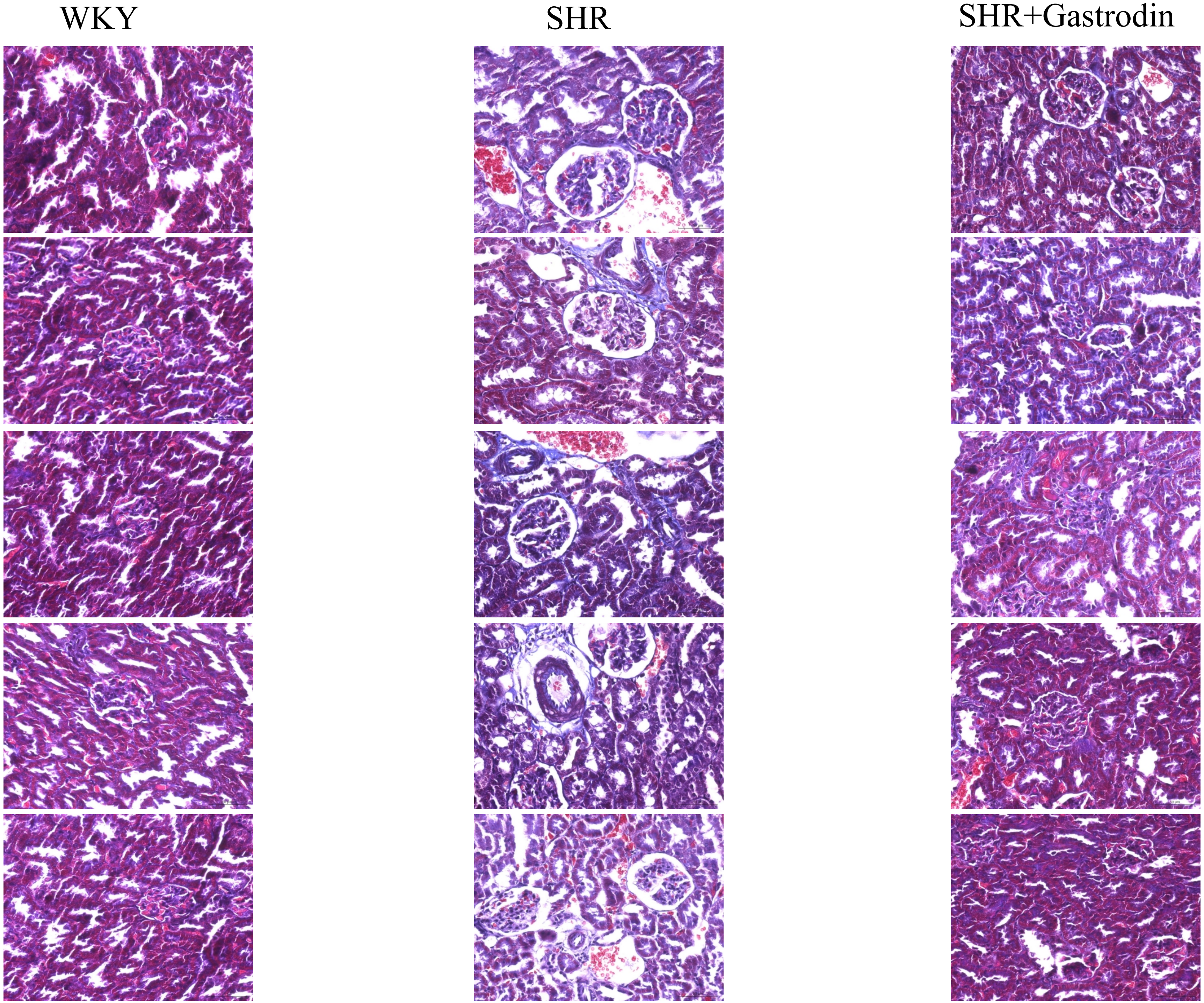

Figure1 E

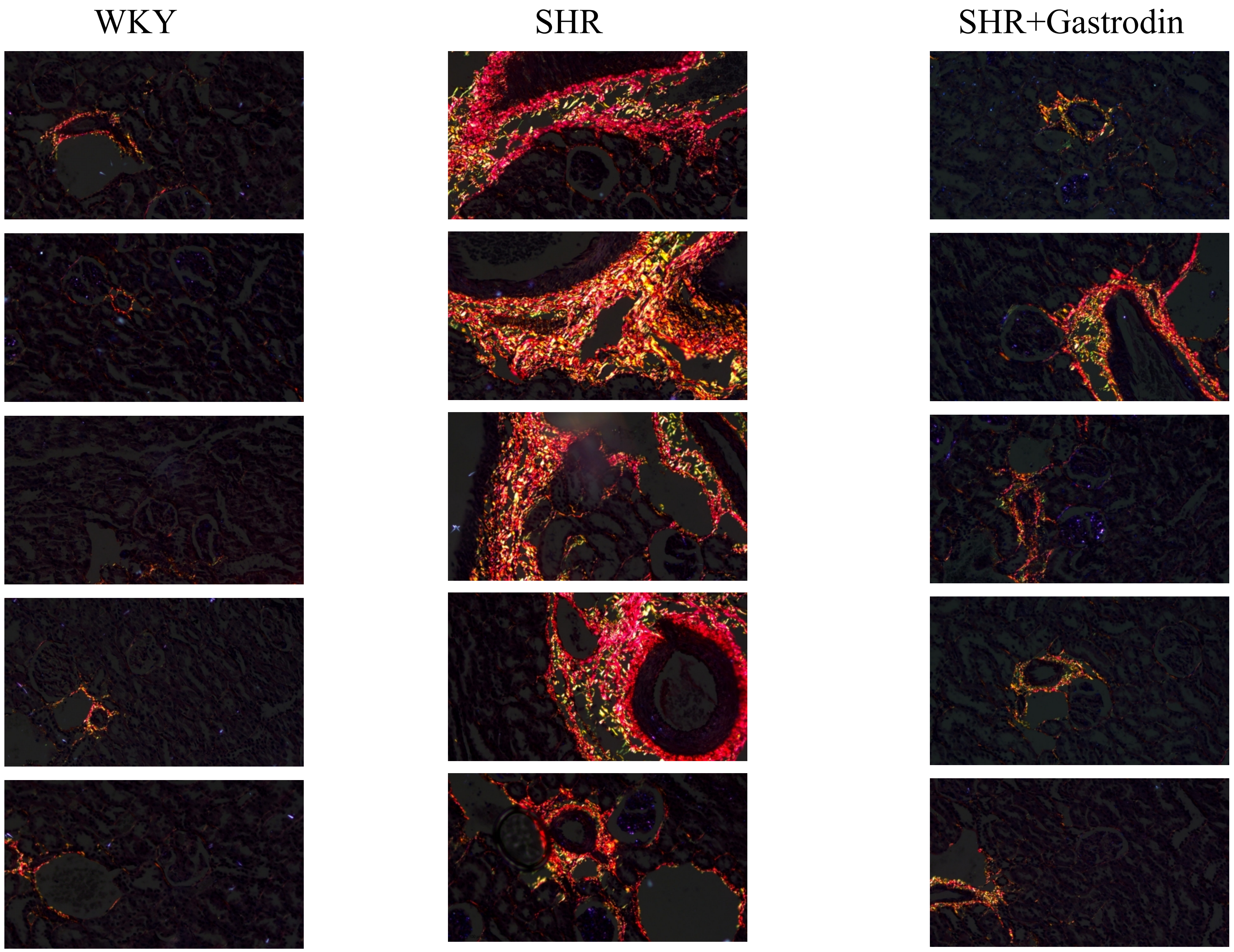

Figure2 A

a-SMA

WKY SHR SHR+Gastrodin

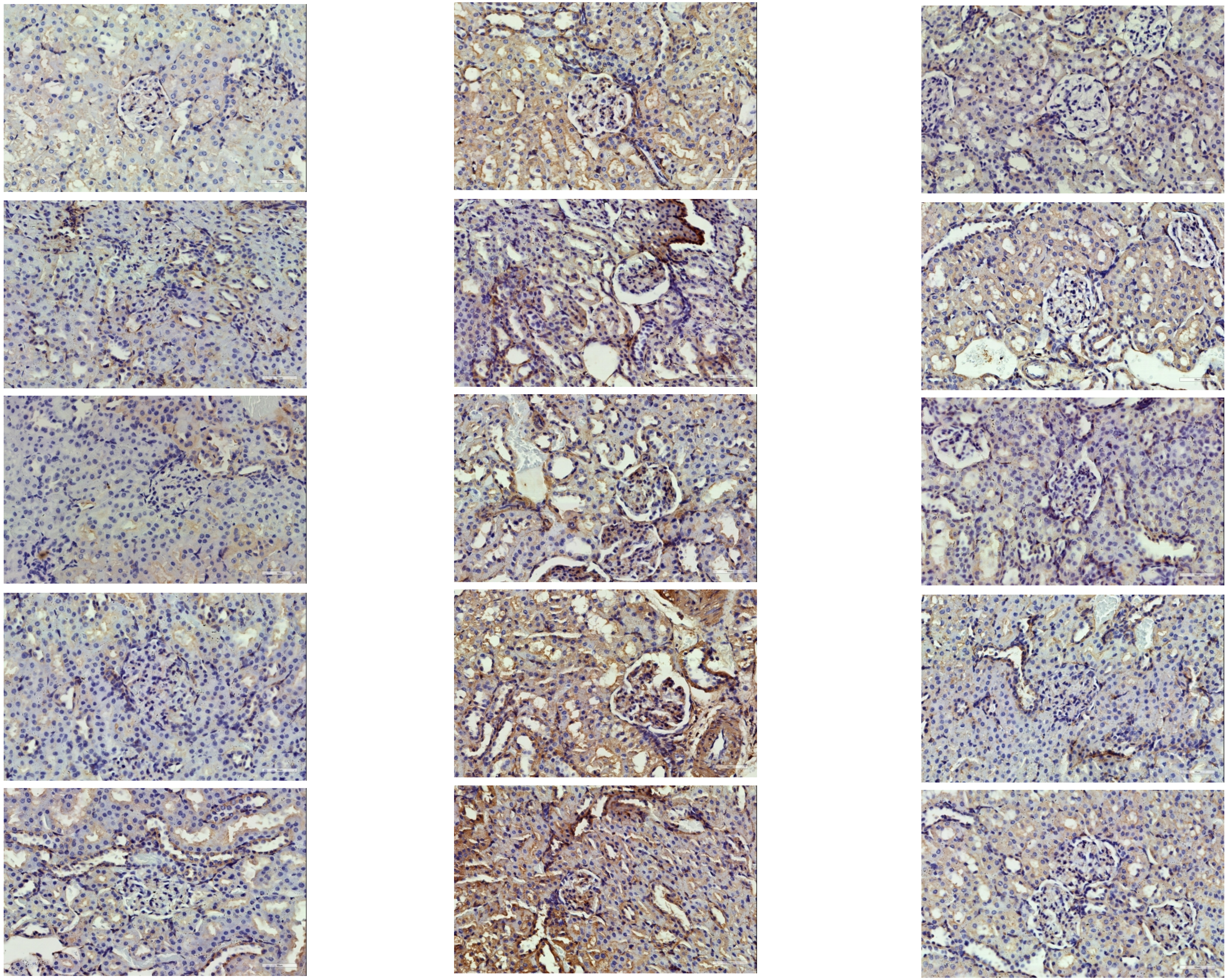

Figure2 C

collagenI

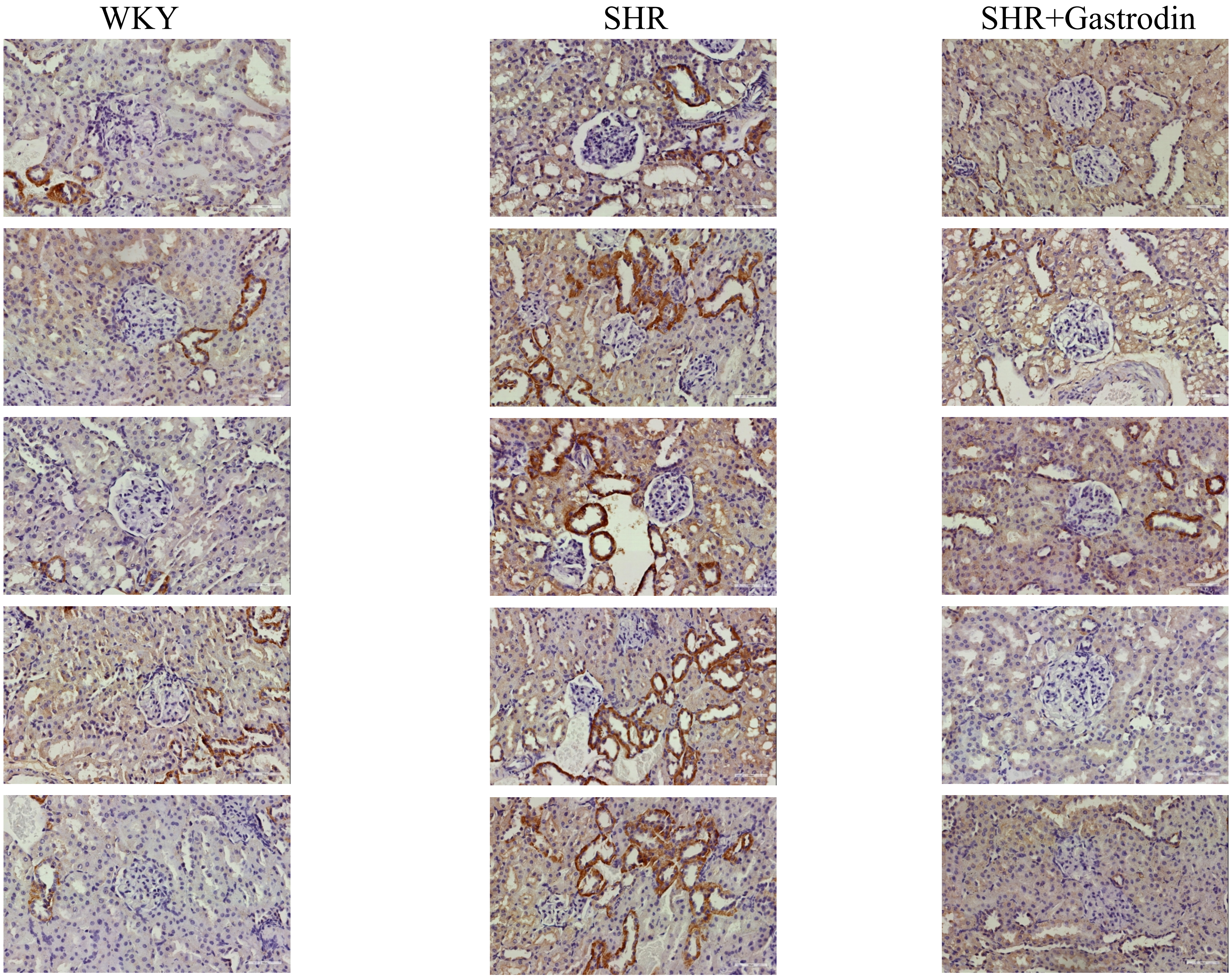

Figure2 E

collagenIII

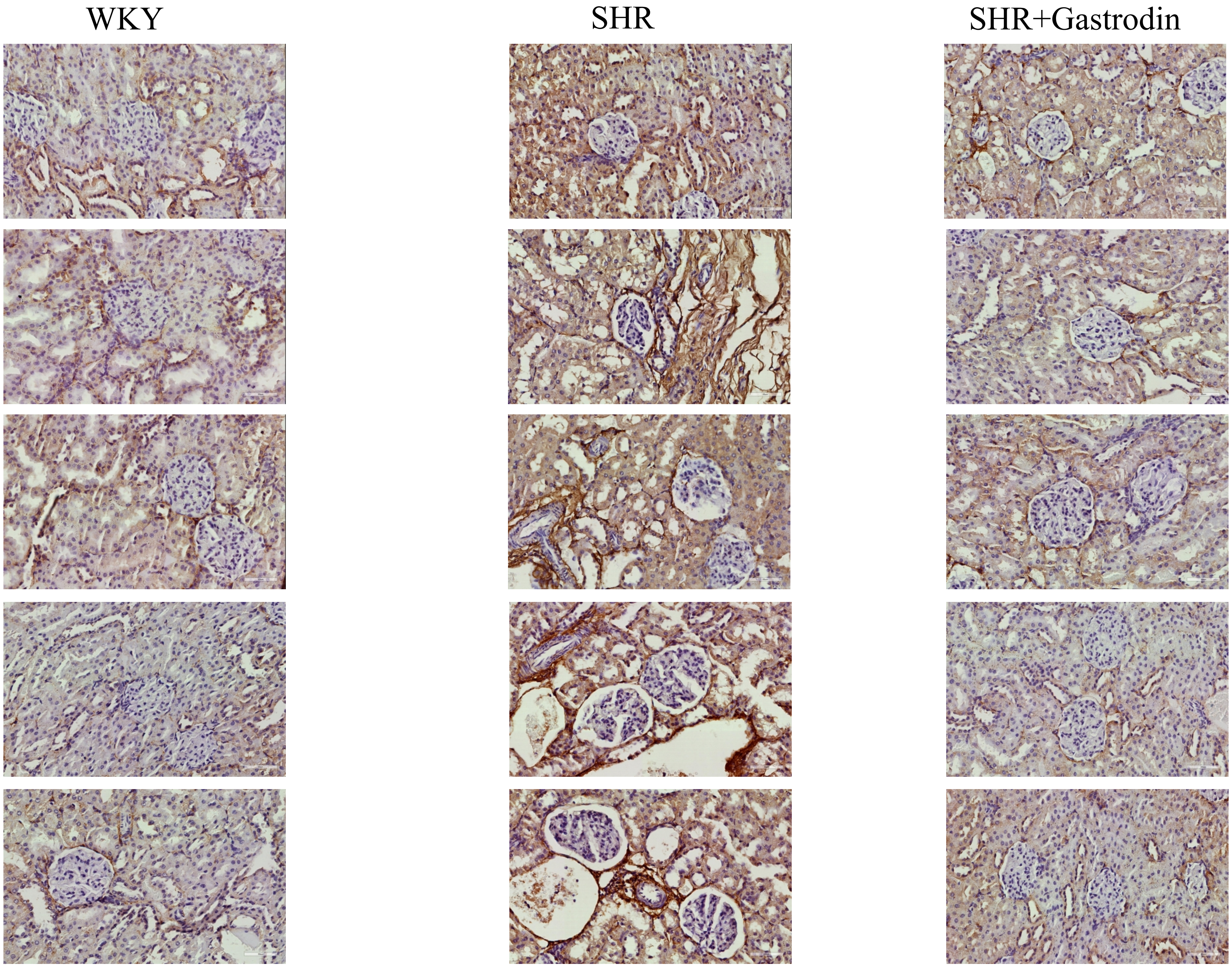

Figure6 A

TGF-β1

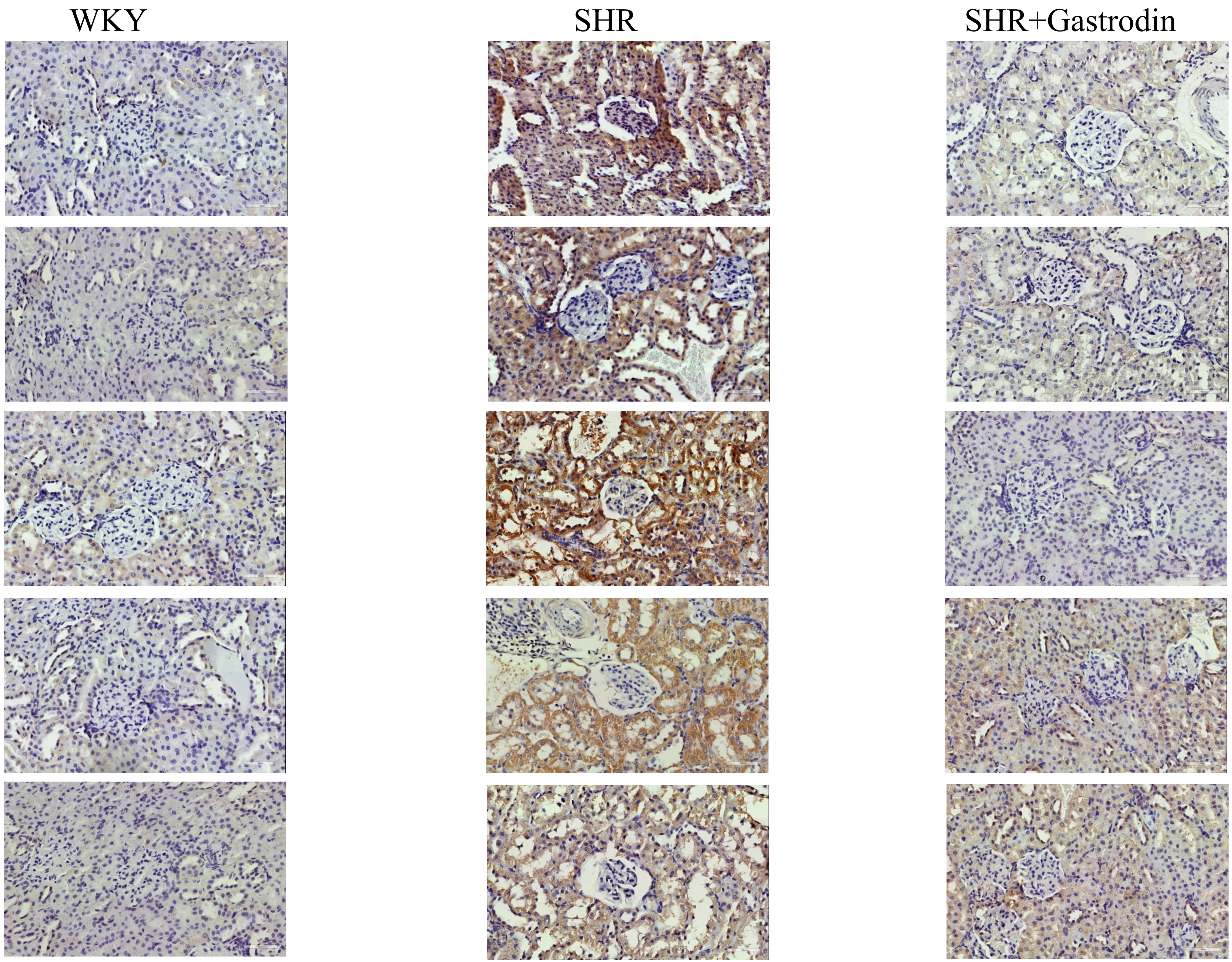

Figure6 C
p-Smad2

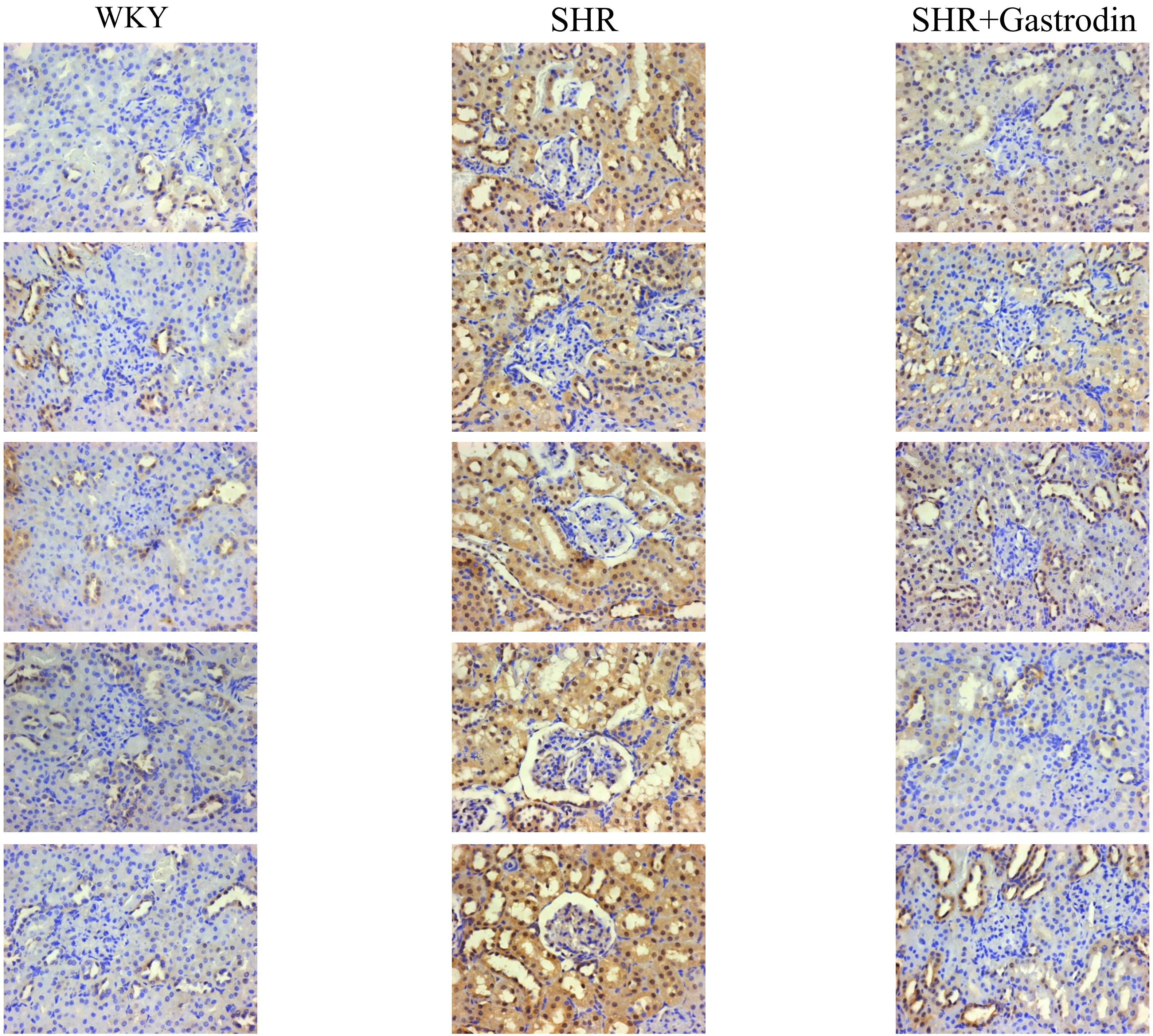

Figure6 C

Smad2

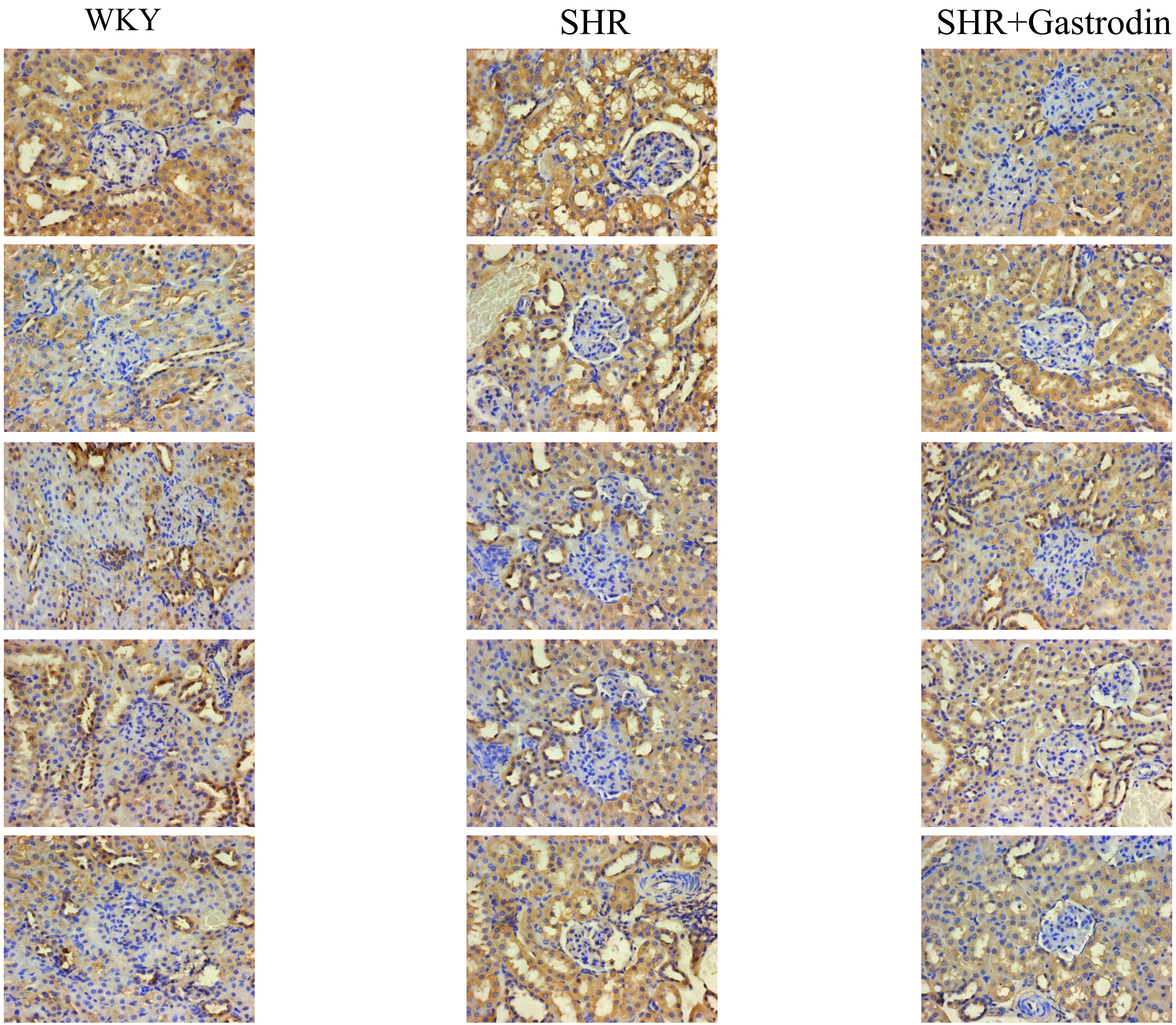

Figure6 E

p-Smad3

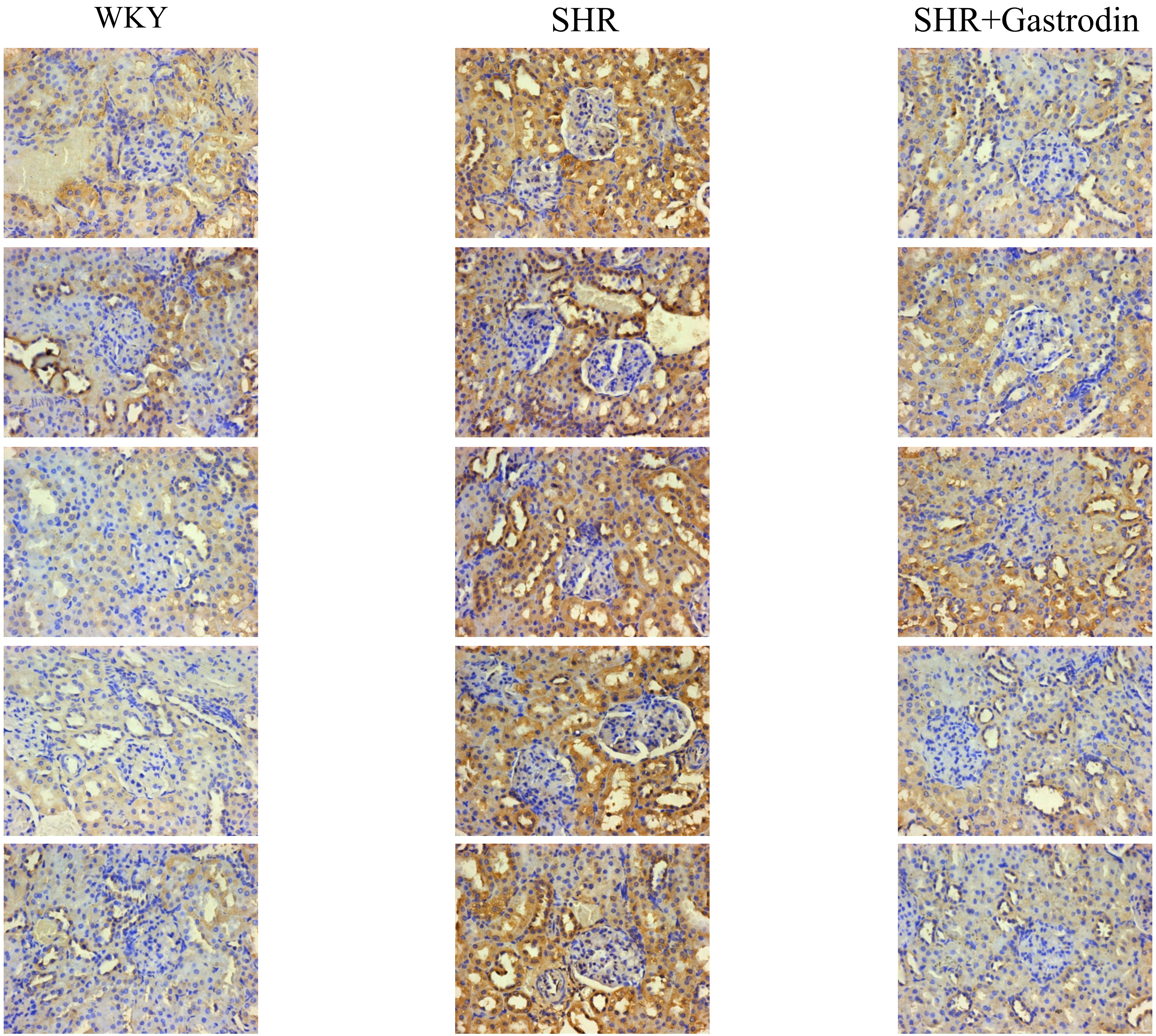

Figure6 E

Smad3

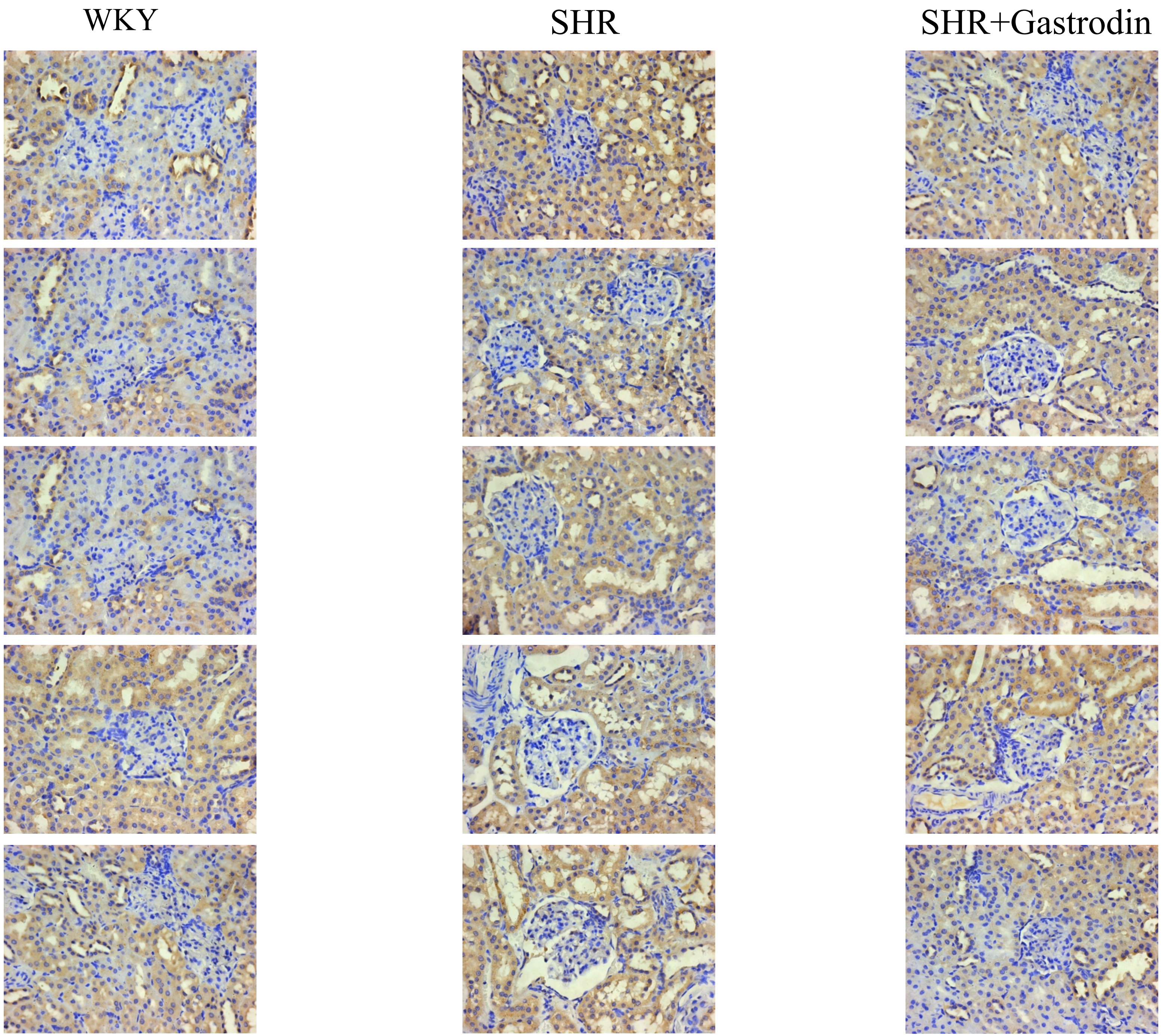

- Figure 7C

**TGF-β1(5ng/ml)**	**-**	**+**	**+**	**+**	**+**
**Gastrodin(μM)**	**-**	**-**	**25**	**50**	**100**

a-SMA

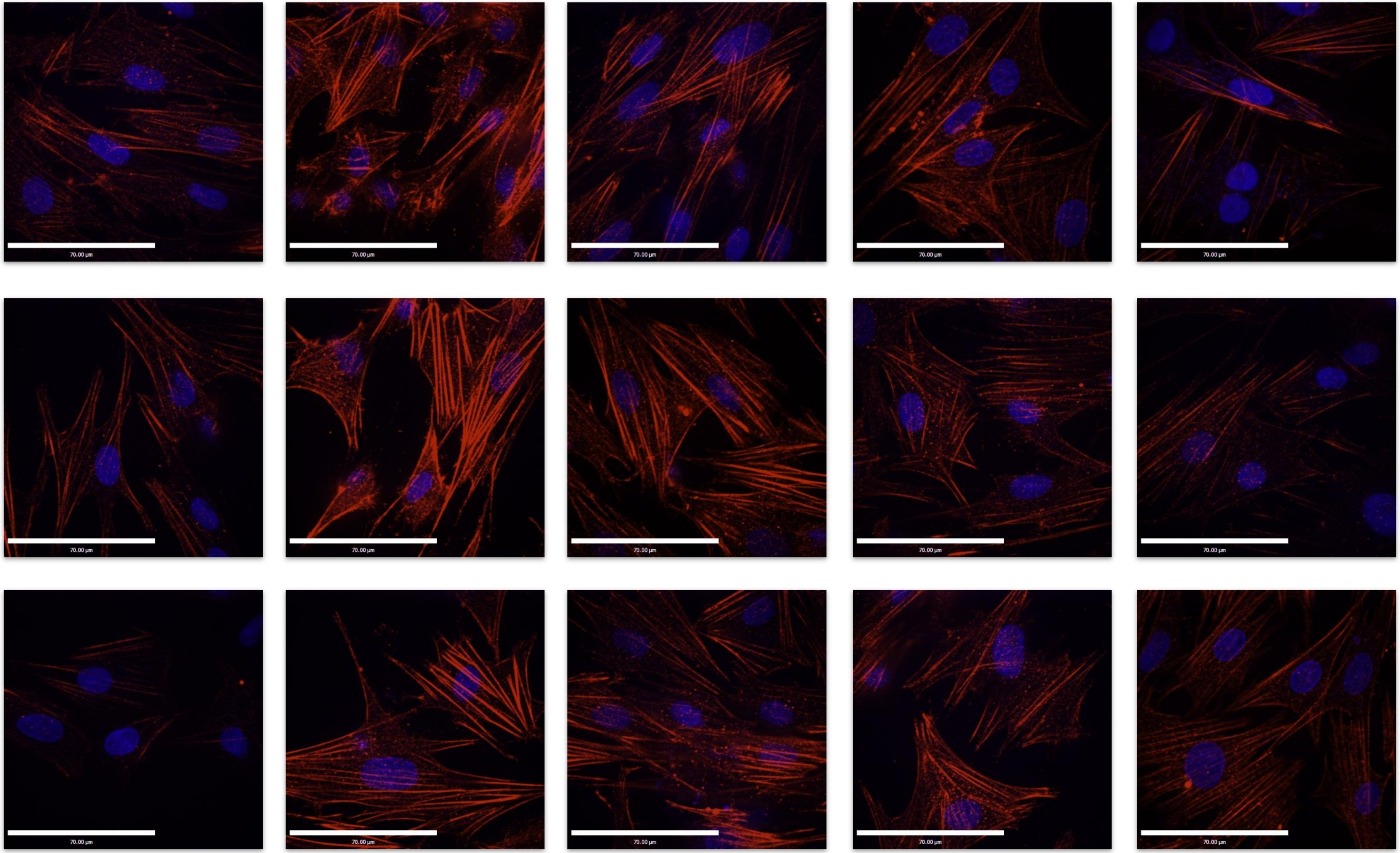

Figure7 C

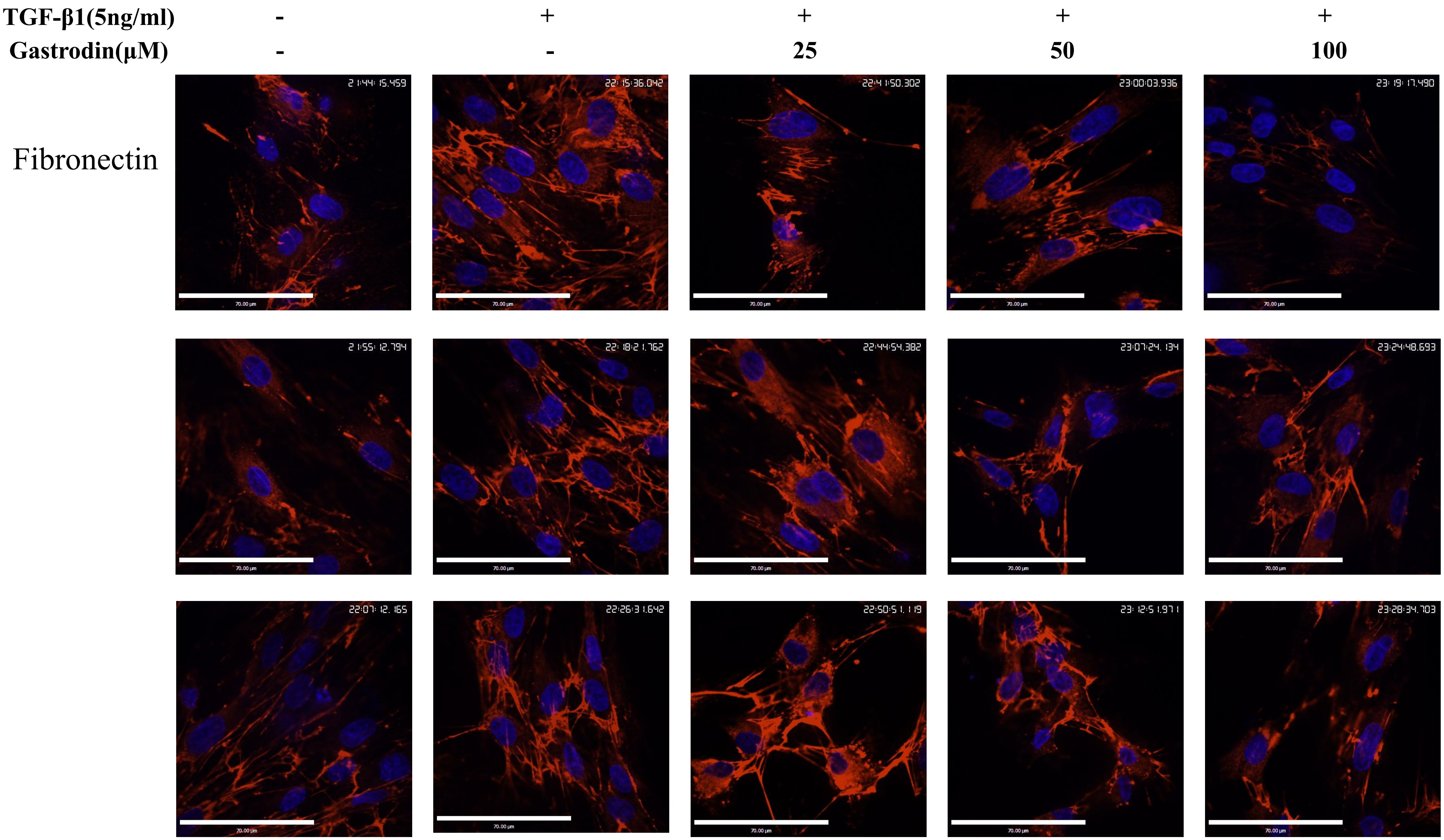

Figure7 D

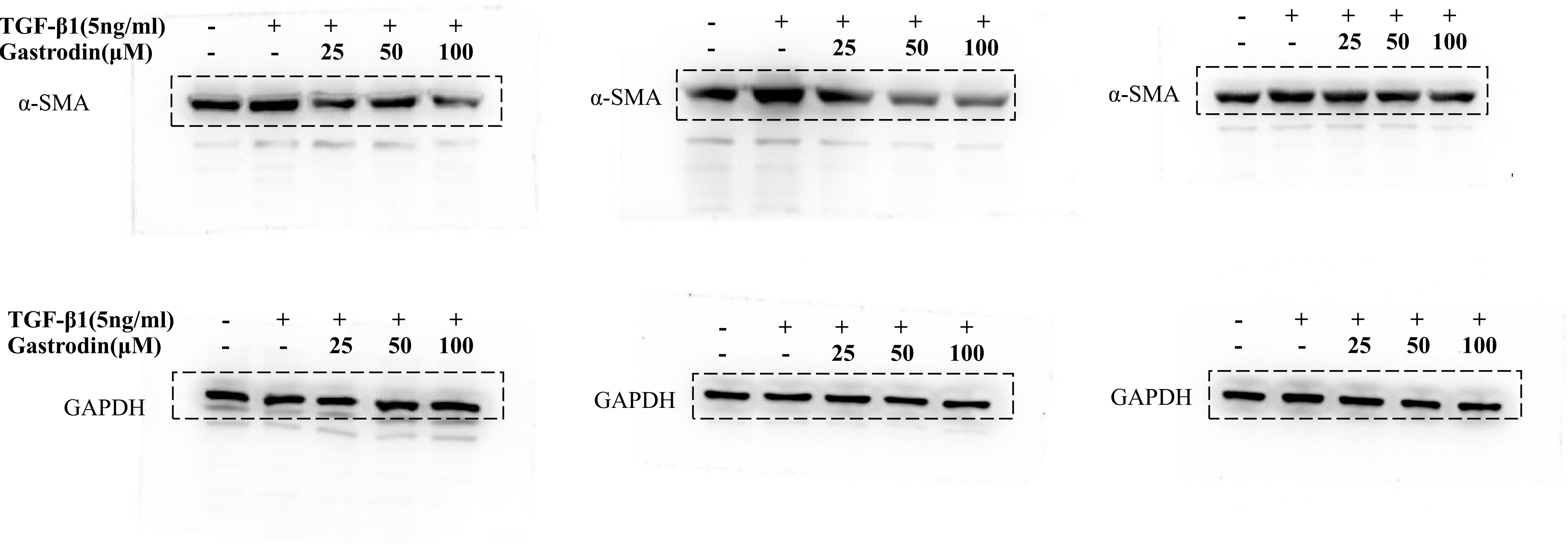
TGF-β1(5ng/ml) - + + + +
Gastrodin(μM) - - 25 50 100
α-SMA
- + + + +
- - 25 50 100
α-SMA
- + + + +
- - 25 50 100
α-SMA
TGF-β1(5ng/ml) - + + + +
Gastrodin(μM) - - 25 50 100
GAPDH
- + + + +
- - 25 50 100
GAPDH
- + + + +
- - 25 50 100
GAPDH

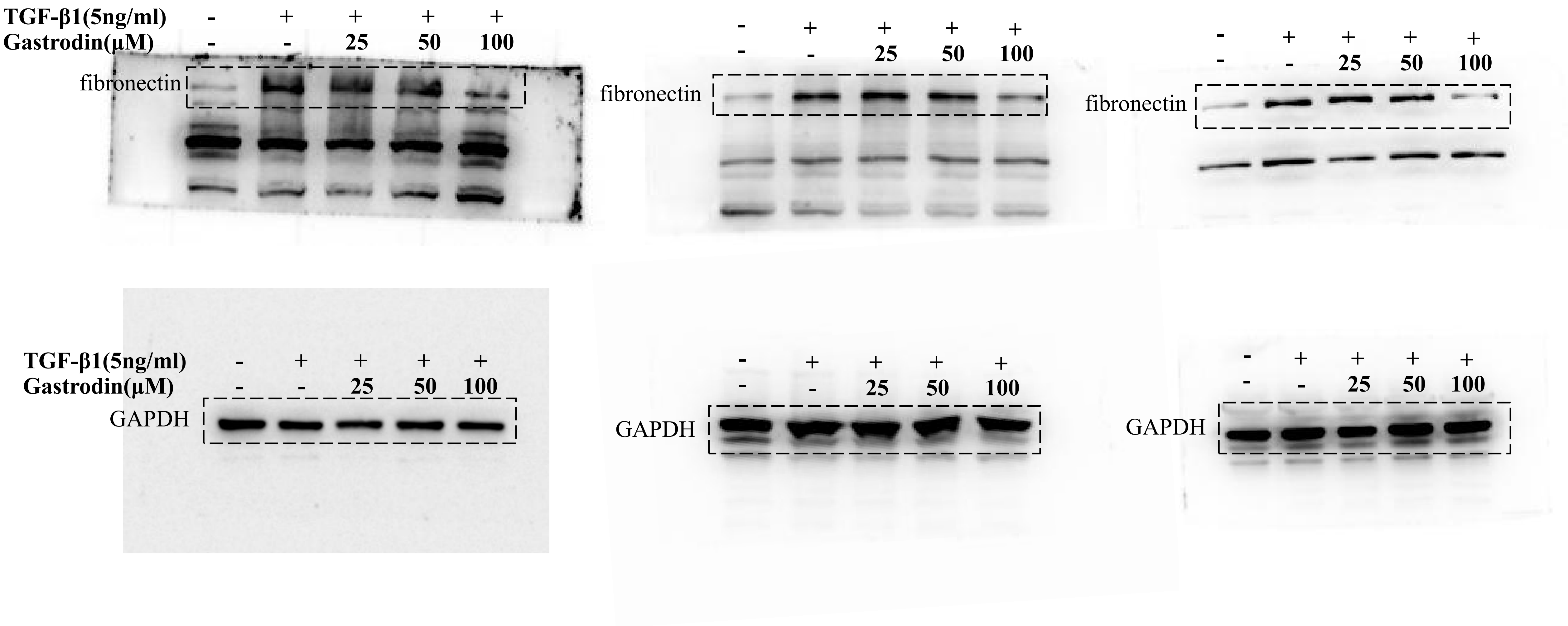
TGF-β1(5ng/ml) - + + + +
Gastrodin(μM) - - 25 50 100
fibronectin
- + + + +
- - 25 50 100
fibronectin
- + + + +
- - 25 50 100
fibronectin
TGF-β1(5ng/ml) - + + + +
Gastrodin(μM) - - 25 50 100
GAPDH
- + + + +
- - 25 50 100
GAPDH
- + + + +
- - 25 50 100
GAPDH

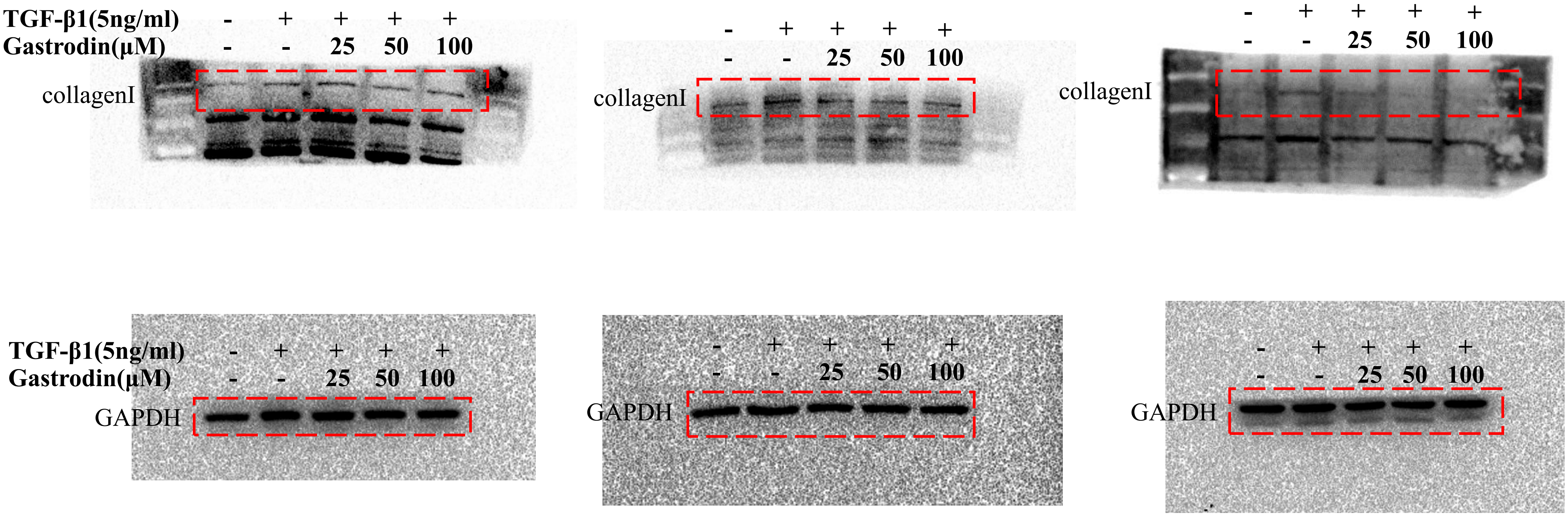
TGF-β1(5ng/ml) - + + + +
Gastrodin(μM) - - 25 50 100
collagenI
- + + + +
- - 25 50 100
collagenI
- + + + +
- - 25 50 100
collagenI
TGF-β1(5ng/ml) - + + + +
Gastrodin(μM) - - 25 50 100
GAPDH
- + + + +
- - 25 50 100
GAPDH
- + + + +
- - 25 50 100
GAPDH

Figure8 A

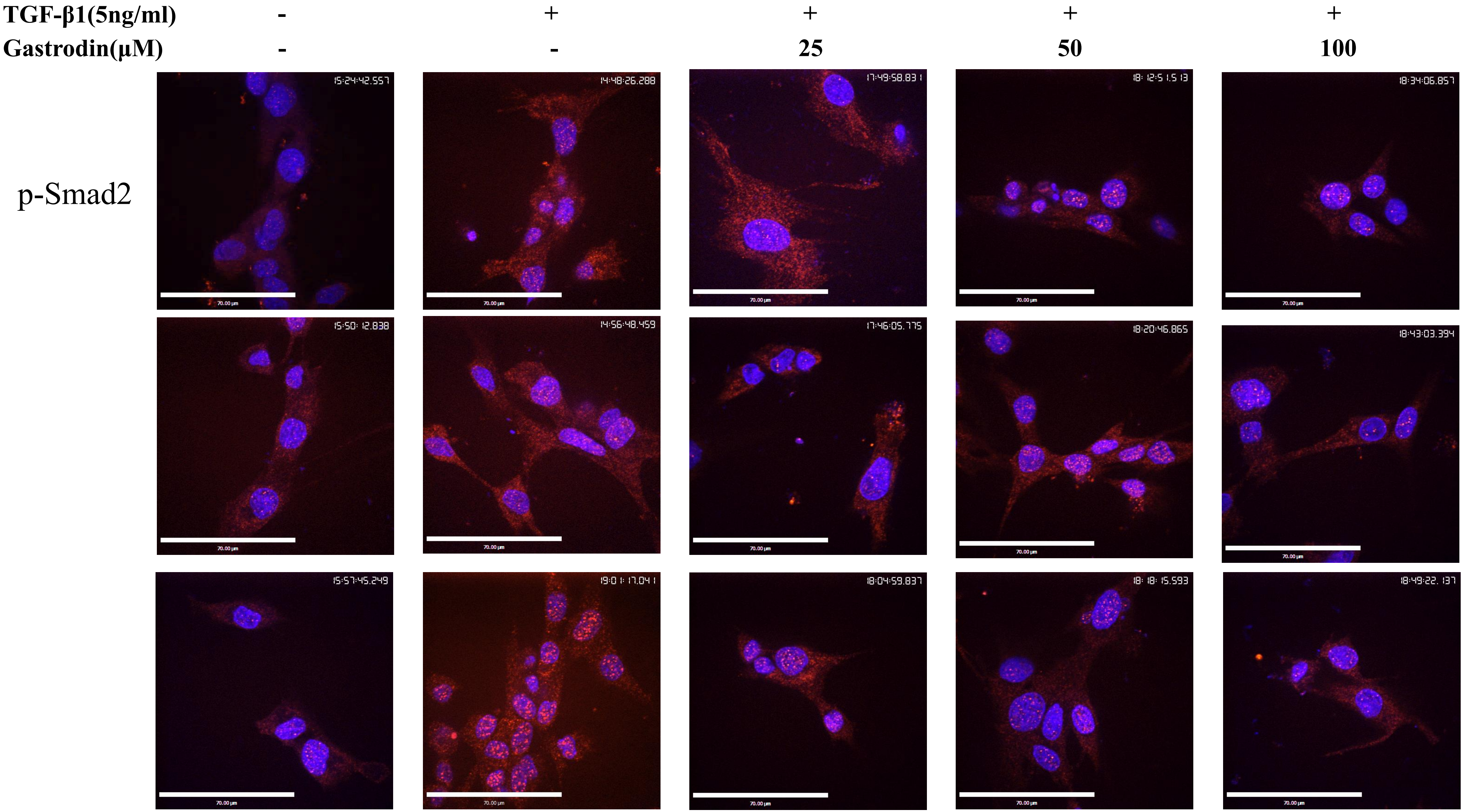

Figure8 A

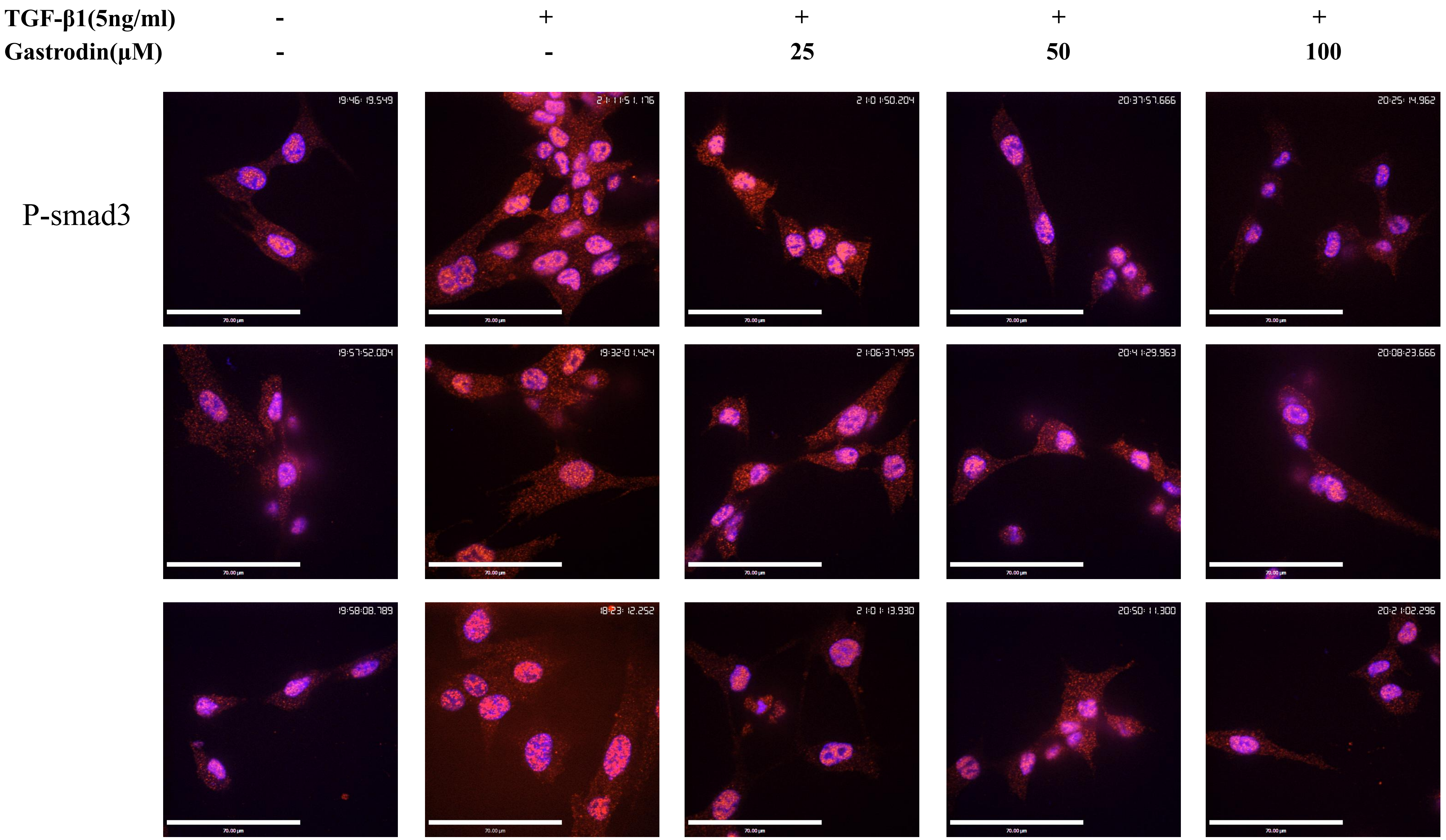

Supplement: Supplementary file 1 [file DataSheet2.PDF]
