# Supplementary figures and images for: Gastrodin attenuates renal injury and collagen deposition via suppression of the TGF-β1/Smad2/3 signaling pathway based on network pharmacology analysis
Source: Front Pharmacol. 2023 Jan 17;14:1082281. doi: 10.3389/fphar.2023.1082281 (PMC9887022; doi:10.3389/fphar.2023.1082281)

- Figure1

**A**

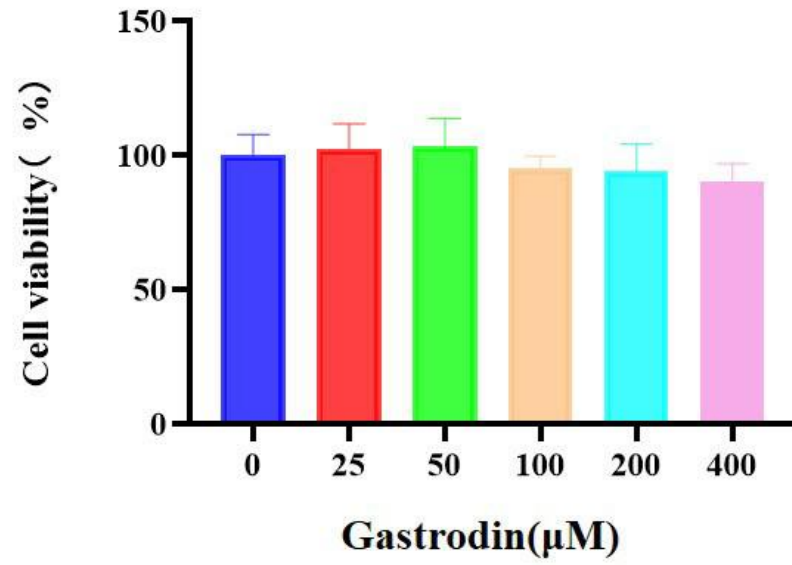

**B**

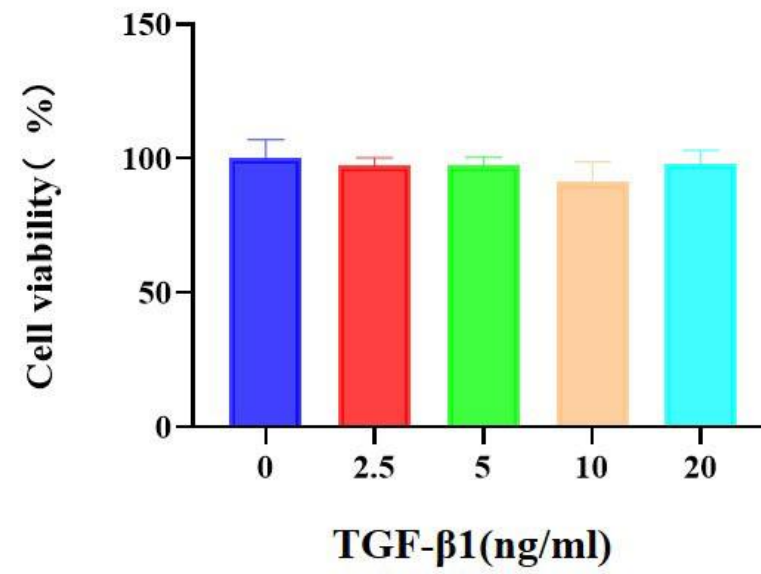

Supplement: Supplementary file 6 [file DataSheet1.PDF]
